# Biomaterials for the Delivery of Growth Factors and Other Therapeutic Agents in Tissue Engineering Approaches to Bone Regeneration

**DOI:** 10.3389/fphar.2018.00513

**Published:** 2018-05-29

**Authors:** Christine J. Kowalczewski, Justin M. Saul

**Affiliations:** ^1^U.S. Army Institute of Surgical Research, San Antonio, TX, United States; ^2^Department of Chemical, Paper and Biomedical Engineering, Miami University, Oxford, OH, United States

**Keywords:** RNA interference, bone morphogenetic protein 2 (BMP-2), collagen, keratin, gelatin, fibrin, chitosan, regenerative medicine

## Abstract

Bone fracture followed by delayed or non-union typically requires bone graft intervention. Autologous bone grafts remain the clinical “gold standard”. Recently, synthetic bone grafts such as Medtronic's Infuse Bone Graft have opened the possibility to pharmacological and tissue engineering strategies to bone repair following fracture. This clinically-available strategy uses an absorbable collagen sponge as a carrier material for recombinant human bone morphogenetic protein 2 (rhBMP-2) and a similar strategy has been employed by Stryker with BMP-7, also known as osteogenic protein-1 (OP-1). A key advantage to this approach is its “off-the-shelf” nature, but there are clear drawbacks to these products such as edema, inflammation, and ectopic bone growth. While there are clinical challenges associated with a lack of controlled release of rhBMP-2 and OP-1, these are among the first clinical examples to wed understanding of biological principles with biochemical production of proteins and pharmacological principles to promote tissue regeneration (known as regenerative pharmacology). After considering the clinical challenges with such synthetic bone grafts, this review considers the various biomaterial carriers under investigation to promote bone regeneration. This is followed by a survey of the literature where various pharmacological approaches and molecular targets are considered as future strategies to promote more rapid and mature bone regeneration. From the review, it should be clear that pharmacological understanding is a key aspect to developing these strategies.

## Clinical significance and current clinical strategies

Bone tissue is a dynamic system which is continuously being remodeled on a day-to-day basis (Barrett, 2010). Because of this property, the normal healing process typically restores the biological and mechanical function of bone following fracture. Unfortunately, the native healing potential of bone is occasionally insufficient for underlying reasons such as smoking (Patel et al., 2013; Taormina et al., 2014), malnutrition (Alvear et al., 1986), congenital disease (Shah et al., 2012), or large defects resulting from tumor resections (Qu et al., 2015). In addition, healthy individuals sometimes experience the inability for bone healing and a return to normal function due to large defects caused by trauma. Bone defects that surpass a size that can spontaneously heal during the lifetime of the individual are known as defects of critical size or critically-sized defects (Schmitz and Hollinger, 1986; Spicer et al., 2012), and are a specific type of non-union. These critically-sized defects frequently lead to secondary complications including morbidity and functional limitations in patients (Tseng et al., 2008). It is estimated that 10% of bone fractures in the United States result in impaired or incomplete healing known as delayed union and non-union, respectively (BMUS, 2014). Lack of osteogenic cells, signaling molecules, and osteoconductive matrix as well as inadequate vascularity all contribute to the inability of bone to heal in these situations (Harwood et al., 2010).

Clearly, bone defects leading to non-union substantially impair activities on a daily basis and impair quality of life. In order for a large defect or other non-union to be functionally restored, a surgical intervention and placement of a bone graft at the injury site is required to attempt to bridge the defect area. Bone grafts are currently the second most common type of tissue transplant in the United States with an estimated 500,000–1.5 million bone-grafting procedures performed yearly. As such, the market in the U.S. alone is ~$1.6–$2.5 billion (Cutter and Mehrara, 2006; Bishop and Einhorn, 2007). Thus, from both a quality of life and an economic perspective, bone grafts that lead to functional restoration following fracture are beneficial.

According to the American Academy of Orthopedic Surgeons the “ideal bone graft” should be “biocompatible, bioresorbable, osteoconductive, osteoinductive, structurally similar to bone, easy to use, and cost-effective” (Greenwald et al., 2001). Osteoconductive materials support bone healing through vascularization, architecture, chemical composition (e.g., calcium sulfate, calcium phosphate, calcium hydroxyapatite, etc.), and surface charge (Urist, 1980). Osteoinduction refers to the process that supports the migration and proliferation of mesenchymal stem cells as well as promoting differentiation of preosteocytes (Urist and Strates, 1971). When designing biomaterials for bone applications a number of factors (i.e., composition, porosity, mechanical properties) must be considered in order to recapitulate native bone. Structural and mechanical property differences between cortical and cancellous bone can be quite significant and must be considered when applying engineered bone substitutes to specific locations. For example, cortical porosity ranges from 5 to 10% while cancellous bone porosity ranges from 75 to 90%. The pore size of areas of native cortical and cancellous bone also differ dramatically (10–600 um; Polo-Corrales et al., 2014); however, it is suggested that the optimal range lines between 200 and 350 um (Murphy et al., 2010; Guda et al., 2014). The scaffold pore size and shape has been shown to significantly modulate osteogenesis (Hulbert et al., 1970; Sanzana et al., 2014) while a heterogeneous distribution of pore size enhances osteogenic potential (Woodard et al., 2007; Di Luca et al., 2016). The mechanical properties of bone also vary widely over orders of magnitude (Polo-Corrales et al., 2014). Taken together, researchers must be vigilant of their scaffold's design and resulting application in the body.

**Figure 1:**
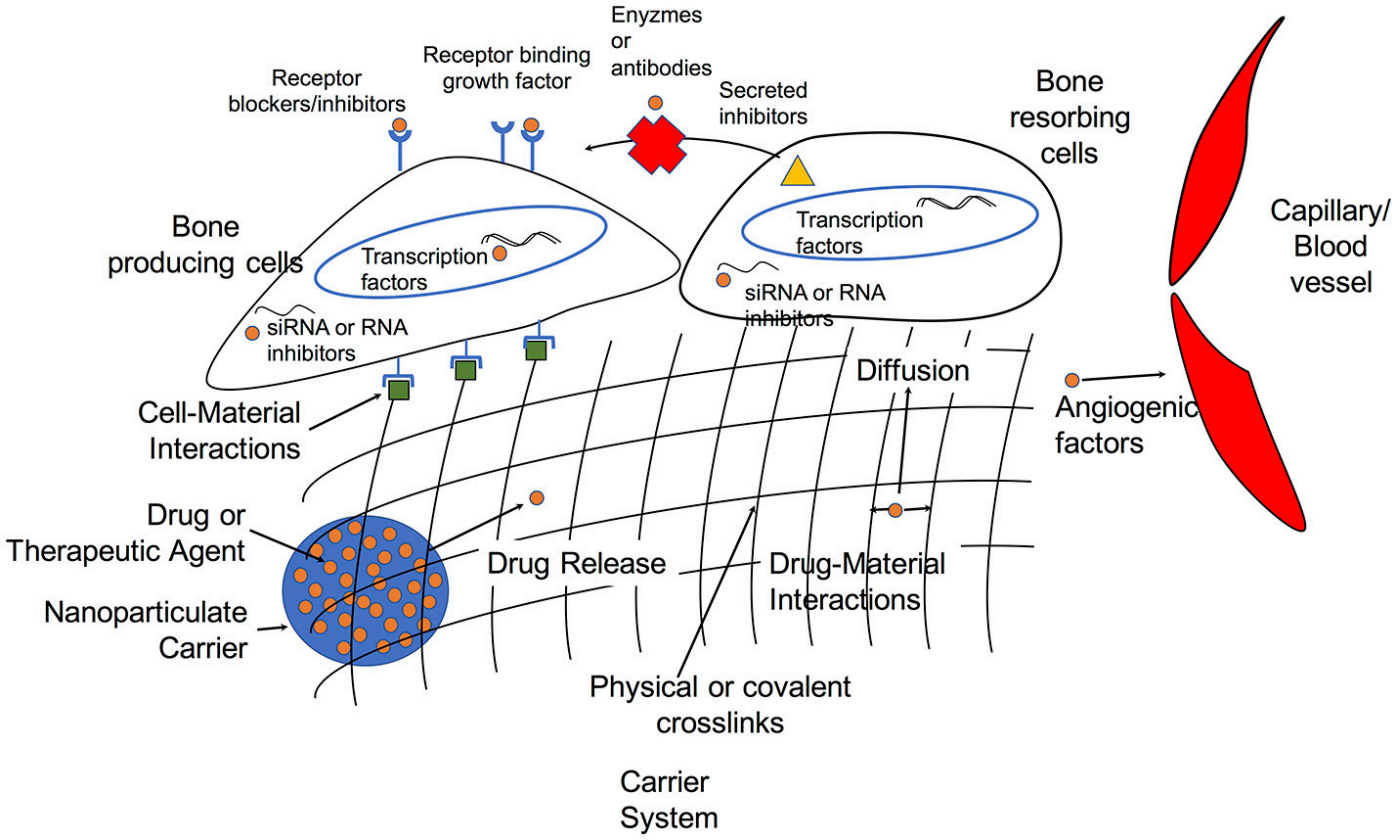
Overview of targets and delivery strategies for growth factors and therapeutic agents in tissue engineering approaches to bone regeneration.

The primary approaches to bone grafting include autografts, allografts, and (more recently) what are referred to as synthetic bone grafts. Below, we briefly discuss some of the drawbacks of autografts and allografts that have led to the push for “off-the-shelf” synthetic bone grafts and some of the on-going needs in the development of these constructs. We then identify existing and promising strategies to improve bone regeneration via pharmacological approaches, and these strategies are summarized in Figure 1.

### Autografts, allografts, and xenografts

Autologous bone grafts remain the clinical “gold standard” for bone defects because of their osteoconductive and osteoinductive properties. Autografts are most often harvested form the iliac crest or other bone (Greenwald et al., 2001). Even though autograft transplantation does not lead to immunogenic response, it has a limited supply, donor site morbidity, and up to 30% failure rate (Schoelles et al., 2005). One solution to overcoming limited supply and donor site morbidity of autologous bone grafts is to use allogeneic or xenogeneic bone sources.

Xenografts are primarily harvested from bovine origins and are osteoinductive/conductive. Research stemming from clinical trials has determined xenografts, primarily from bovine origins, to be unsuitable due to increased risk of infection, immunogenicity, and host rejection (Trice, 2009; Mehta et al., 2012).

Allografts harvested from cadaveric sources have the advantage of being osteoconductive and osteoinductive while being available through regional tissue banks in a variety of sizes and shapes. Again there can be limited supply, and allograft treatments are associated with risk of rejection and transmission of infectious disease (Grover et al., 2011). While tissue processing and sterilization through freezing or 25 kGy gamma irradiation virtually eliminates the possibility of disease transmission, irradiation causes a number of adverse effects on tissue properties such as weakening (Nguyen et al., 2007).

Demineralized bone matrix (DBM) products derived from allogeneic sources once comprised ~50% of the bone graft market (Cutter and Mehrara, 2006) but since the emergence of a number of FDA-approved synthetic bone grafts (see below), DBM now accounts for ~20% of the grafting market (Gruskin et al., 2012). The osteogenic potential of commercial DMB, however, is highly variable due differences in the amounts of osteogenic molecules (e.g., BMP-2 and BMP-7; Bae et al., 2010) likely due to variability between donors, sterilization techniques, and storage methods (Wang et al., 2007). Non-standardized production and lack of precise control conditions needed for storage to maintain bioactivity has resulted in inconsistent results with different products as well as product lot numbers (Wang et al., 2007; Gruskin et al., 2012). In essence, although the constituents are known, DBM is an undefined material. For this reason, the use of synthetic bone grafts as a tissue engineering approach has become an important consideration for clinical treatment of bone defects and other orthopedic applications.

### Biomedical materials as bone fillers

In the next section, the use of what are referred to as synthetic bone grafts are discussed. As used in the literature, “synthetic bone graft” is a bit of a misnomer. A synthetic bone graft need not be synthetic. More specifically, collagen is the most commonly used material component. Synthetic bone grafts also rely on the use of osteogenic molecules to promote bone growth.

Here, however, we briefly discuss some biomedical materials that have been used as bone grafts, and in this context they are often referred to as bone fillers. Common advantages to the use of such materials are that they, like synthetic bone grafts, are off-the-shelf products that can be manufactured in a repeatable manner and in sufficient quantities. These materials may or may not be osteoconductive and while they can promote bone tissue formation, they do not have the same degree of osteoinductive or osteogenic behavior as molecules such as bone morphogenetic protein 2 (BMP-2). A common feature, though, among these materials is they are traditional biomedical devices that do not achieve their primary function through chemical action (i.e., they are not drugs).

#### Ceramics

Examples of ceramic bone fillers include Bioglass, calcium phosphate, calcium sulfate, and hydroxyapatite. In general, an advantage of ceramic materials is their similar material and mechanical properties to the mineral composition of bone. Indeed, hydroxyapatite is the main inorganic constituent of bone, so its use as a biomedical material is logical.

More recently, ceramics have been used as composites with natural polymers in an effort to obtain the beneficial properties of each material. For example, Bioglass in conjunction with gelatin led to mechanical strength but with increased pore size (Aksakal and Demirel, 2017). Likewise, the use of Bioglass with a tricalcium phosphate/alkylene oxide copolymer or tricalcium phosphate have been used in a large animal model as a moldable material for conformation to defect geometry to promote both intraosseous and intramuscular bone formation (Barbieri et al., 2017). Ceramic materials can also be used for the delivery of antibiotics to prevent infection (see further below on synthetic polymers) (Kanellakopoulou and Giamarellos-Bourboulis, 2000; Li and Chang, 2005).

#### Polymeric materials

One of the most well-known bone filler materials is poly(methylmethacrylate) or pMMA, also known as bone cement. pMMA is a non-biodegradable material and can be considered a bone filler in the truest sense of the word. That is, a major drawback to the use of pMMA is its non-degradable nature, which is known to impede bone remodeling (Freeman et al., 1982; Jensen et al., 1991), likely through an unfavorable cellular microenvironment (Maloney et al., 1990). Like other polymers pMMA also suffers from poor mechanical properties (Saha and Pal, 1984). As noted above for some ceramic materials, one clinical use for synthetic polymers such as pMMA is for the delivery of antibiotics to prevent infection. A significant challenge for fractures requiring open reduction is bacterial infection (Seekamp et al., 2000). As such, the ability to deliver gentamicin (Klemm, 1993; Shi et al., 2006), vancomycin (Zelken et al., 2007; Li et al., 2010), and other antibiotics (Efstathopoulos et al., 2008; Shi et al., 2010) is often of considerable importance.

Biodegradable natural and synthetic polymers have been used as bone fillers, but their mechanical properties are not advantageous. Synthetic biodegradable polymers and natural polymers are widely used for synthetic bone grafts at the clinical and pre-clinical level, as discussed in the following section. However, a growing use for biodegradable polymers such as poly(glycolic acid) (PGA), poly(lactic acid) (PLA), copolymers of PGA and PLA (PLGA), polycaprolactone (PCL) as well as natural and non-degradable polymers is in 3-D printing technology (Taboas et al., 2003; Hollister et al., 2005, 2015). 3-D approaches allow for the presentation of well-defined pore sizes and organizational structures that can be optimized to promote favorable cellular response.

To date, bone formation obtained by bone fillers such as those described above simply do not achieve the levels of bone formation obtained from autografts. This unmet need has led to investigation of the use of the synthetic bone grafts that make use of some of the principles of tissue engineering such as knowledge of the growth factors responsible for bone formation.

### Synthetic bone grafts: a tissue engineering approach to bone regeneration

Tissue engineering can be considered to consist of the triad of biomaterial scaffolds, cells, and signals (either chemical/molecular or physical/mechanical; Zerhouni, 2005). To address the disadvantages of ceramic and polymer-based bone substitutes, tissue engineering techniques have been applied to develop a number of growth factor-based products. These approaches typically consist of a collagen sponge as the biomaterial and recruit endogenous cells to the defect site. These strategies also consist of molecular signals in the form of growth factors/morphogenetic proteins. While bone formation and regeneration is controlled by a cascade of molecules delivered at precise locations and times, only two commercially produced growth factors have been FDA approved for clinical use: bone morphogenetic protein-2 (BMP-2) and bone morphogenetic protein-7 (BMP-7), which is also known as osteogenic protein-1 (OP-1).

BMPs primarily act as cytokines mediating the differentiation of mesenchymal cells into cartilage and bone forming cells (Ebara and Nakayama, 2002). The functions of various BMP molecules have been described both in general (Wang et al., 2014) and in skeletal tissue (Rahman et al., 2015; Wu et al., 2016). In particular, BMP-2 and -7 (OP-1) have been identified as playing critical roles in bone formation and healing by their ability to induce osteoblast differentiation (Spector et al., 2001). The genetic sequence of BMP was first identified in 1988 which subsequently allowed for the commercial production of various BMPs through the use of recombinant gene technology (Wozney et al., 1988). This marked a notable increase in the use of BMPs in bone tissue engineering. BMP-2 and BMP-7 are highly osteoinductive growth factors and their adsorption onto osteoconductive carriers such as collagen is based on difficulties associated with the efficacy of solubilized rhBMP-2 such as a short half-life (Yamamoto et al., 2003) and up-regulation of receptor-binding antagonists (Ebara and Nakayama, 2002).

Given that collagen is the main organic component of bone, is osteoconductive, and can support mineralization and cell ingrowth it is reasonable that DBM (Niederwanger and Urist, 1996) or absorbable collagen sponges (ACS) (Nevins et al., 1996) were among the first carriers for BMP-2. Absorbable collagen sponges (ACS) and collagen particles were the first generation of tissue-engineered products on the market with FDA approval (McKay et al., 2007). Initial clinical trials conducted with rhBMP carriers such as collagen for spinal fusion applications were not only successful but outperformed autologous bone grafts (Schimandle et al., 1995).

Like many first-generation products there have been a number of drawbacks to rhBMP-2 collagen carriers. Positioning of the ACS is often difficult and secondary displacement of the collagen sponge has been reported (Schmidmaier et al., 2007). Collagen is quickly degraded *in vivo*, causing voids to develop within the new bone matrix due to the inability of collagen to provide a structure which lasts long enough for cell migration (Friess et al., 1999). Perhaps the main issue associated with the ACS is an initial burst release of rhBMP-2 into the local environment, leading to heterotrophic ossification (Brown et al., 2011). Another side effect of this burst release is the activation of osteoclasts in the surrounding environment at high BMP-2 concentrations (Okamoto et al., 2006; Suliman et al., 2015), which may lead to bone resorption. Surrounding mesenchymal lineage progenitor cells in adjacent musculature also receive rhBMP-2 dosage, leading to differentiation into osteoblasts and subsequent mineral deposition in muscle tissue (Katagiri et al., 1994).

This burst release is a cause for concern since the current clinical modalities use 10- to 1,000-fold higher concentrations than that of native BMP-2 levels found in the body (Vaibhav et al., 2007). Therefore, novel methods of delivery and also alternative molecular targets continue to receive attention at the pre-clinical level. Below, we discuss several classes and types of alternative carrier systems being investigated. We then consider therapeutic strategies beyond the existing rhBMP-2 paradigm.

## Alternative biomaterial carriers for molecules promote bone regeneration

Great effort has been placed on developing biomimetic scaffolds which provide a three-dimensional matrix for cell migration and proliferation. Although modern biomaterials lack the temporal and spatial complexity to fully mimic the native extracellular matrix (ECM) (Daley et al., 2008), they capture many of the essential elements. Through their mechanical, material, and chemical cues, biomaterials are able to influence a number of cellular functions (e.g., cell-matrix interactions) including proliferation, differentiation, apoptosis, and signaling (Daley et al., 2008). Ideally, these biomaterials should not only be non-toxic but also biodegradable so that the artificial matrix will degrade over time and give way to new tissue being formed at the site where the material was implanted. The degradation/resorption of the scaffold should be tailored to the rate of bone turnover and healing rates of the injured bone. For example, spinal fusion cases can require 9 months whereas craniofacial application are of a shorter (3–6 months) duration (Bose et al., 2012). For this reason, we focus here on engineered degradable polymers which are capable of degradation rates ranging from weeks to months.

In addition to their role in mimicking the structural aspects of the ECM, biomaterial carriers can also be used to promote the controlled release of therapeutic agents. Bioactive molecules and therapeutics can be incorporated or physically entrapped within the polymer network. One important design parameter to regulate carrier degradation as well as rhBMP-2 release is to modify the mesh size of the polymer network through the crosslink density. When the overall crosslink density of a hydrogel network is increased, it decreases the molecular distance between crosslinks, resulting in a smaller hydrogel mesh size (Lowman and Peppas, 1999). This in turn lowers the diffusivity of the therapeutic agent, allowing tunable rates of release to be achieved by varying the crosslink density of a hydrogel (Saltzman, 2001). This is one approach used in many hydrogel systems since it is the simplest method for successful encapsulation of rhBMP-2. The use of electrospinning nanofibers in which the growth factor is embedded within the fibers employs a similar strategy (Li et al., 2006). A burst release is often observed (Huang and Brazel, 2001) followed by release of the therapeutic agent via some combination of diffusion and material degradation. In cases where there is minimal interaction between the therapeutic agent and the carrier, first order release is common (Saltzman, 2001).

While hydrophobic, van der Waals, and electrostatic interactions between the material and growth factor as well as entrapment in the polymer network are inherently present, additional molecules can be introduced into the biomaterial to retard the rate of release. Examples of these immobilization methods include affinity binding such as heparin-binding (Yang et al., 2010, 2012; Jeon et al., 2011; Wang et al., 2013), ionic interactions such as those provided by chondroitin sulfate (Wang Y. et al., 2010; Bae et al., 2012), cyclodexterins (Del Rosario et al., 2015), protease degradable tethers (Tokatlian et al., 2010), succinylation (Tsujigiwa et al., 2006), alkylation (Tachibana et al., 2006; Han et al., 2015), and even covalent conjugation (Shen et al., 2009; Zhang et al., 2010).

In addition to direct modification of the material, rhBMP-2 can be encapsulated via micro, macro, and nanoparticles alone (White et al., 2013) or within these particles embedded within a scaffold (Park et al., 2009). In this way, rhBMP-2 release is regulated by the rate of particle degradation. When rhBMP-2 microspheres are entrapped in a hydrogel network they retain rhBMP-2 for significantly longer periods of time (compared to adsorption) resulting in prolonged bioactivity (Kempen et al., 2008). Synthetic polymers are commonly used for this purpose due to their predictable rates of degradation and release (Brown et al., 2011; Hernandez et al., 2012). Natural polymers such as chitosan (Niu et al., 2009; Hou et al., 2012; Cao et al., 2014), alginate (Abbah et al., 2013), and gelatin (Patel et al., 2008) are being increasingly used for rhBMP-2 microsphere encapsulation. In summary, examination of key parameters noted above (material network properties, material modification, and encapsulation) associated with rhBMP-2 release has allowed for the development of systems that approach zero-order release in some cases (Pillay et al., 2005).

### Synthetic materials for bone regeneration

Synthetic polymers have the advantage of being able to be manufactured in large quantities and customizable to show a wide range of material properties. A number of biodegradable synthetic polymers are already FDA-approved for use in humans. Poly(a-hydroxy acids); poly(lactic acid) (PLA), poly(glycolic acid) (PGA), and poly(lactic-co-glycolide) (PLGA) (Sokolsky-Papkov et al., 2007) have been used for decades in orthopedic applications (Puska et al., 2011; Razak et al., 2012). These polymers undergo bulk degradation by hydrolysis of which the rate of degradation can be tailored to the demands of the tissue. Their degradation products, lactic acid and glycolic acid, can be processed and excreted from the body. Unfortunately, these components can decrease the pH of the local environment (Suganuma and Alexander, 1993). Synthetic polymers have been reported to cause immunogenicity and toxicity due to chemical crosslinkers and polymerizers used in their production (Athanasiou et al., 1996; Williams et al., 2005). Many of these polymers, especially the FDA-approved polymers, are hydrophobic and lack cell-binding domains such as the arginine:glycine:aspartic acid (RGD) sequence, thus hindering cell attachment (Pierschbacher and Ruoslahti, 1984; Lieb et al., 2003).

Fortunately, RGD and other cell-binding domains can be incorporated into synthetic materials by incorporation of chemically labile groups in the polymer (Hersel et al., 2003). Synthetics can also be tuned for their rates of degradation (Ifkovits et al., 2009; Qiu et al., 2011), release of therapeutic agents (Ashley et al., 2013), and mechanical properties (Chiou et al., 1996; Martello et al., 2014). These materials can also be mixed with calcium phosphates (e.g., hydroxyapatite) (Pathi et al., 2010) or natural polymers (e.g., collagen) (Ochi et al., 2003; Niu et al., 2011) to enhance cell attachment and osteoconductivity. For synthetic polymers to achieve both osteoconductive and osteoinductive properties they must be a combination product composed of the synthetic polymer, calcium phosphate, a natural polymer or cell binding motif, and osteogenic growth factor (Niu et al., 2009; Lu et al., 2012).

### Natural materials for bone regeneration

Interest in natural polymers has been increasing due to a number of beneficial properties such as low cytotoxicity, favorable degradation byproducts, low immunogenicity, and similarity to the ECM. Living organisms synthesize a number of macromolecular components which a biological environment can recognize and degrade hydrolytically or metabolically (Mano et al., 2007). Polysaccharides (e.g., alginate, chitosan) and proteins (e.g., collagen, silk, fibrin, and keratin) are extracted from renewable plant (Plowman et al., 2010), algae (Percival, 1979), animal (Zhang et al., 2006; Plowman et al., 2010), or human (Mosesson and Sherry, 1966; Reichl, 2009) resources. Here, we briefly discuss several promising carrier systems for rhBMP-2 or other molecular therapeutics as discussed below.

#### Fibrinogen/fibrin

Fibrin is a fibrous protein involved in hemostasis when soluble fibrinogen (340 kDa molecular weight) is polymerized in the presence of the enzyme thrombin activated in response to injury. Orthopedic surgeons have long appreciated the significantly positive effect blood clots (which include fibrin) have on bone regeneration. Even though fibrin is not a regular component of the uninjured ECM, it serves as a temporary matrix for wounded tissue until remodeling can replace it with tissue-specific ECM (Clark et al., 1982). Not only does fibrin provide structure, but the material alone has been reported to induce osteogenic differentiation (Martino et al., 2009) and angiogenesis (Feng et al., 2013). Thrombin-mediated release of fibrinopeptides A and B (Profumo et al., 2003) attracts inflammatory cell migration to the site of injury (Senior et al., 1986) and induces cell proliferation (Sporn et al., 1995). Fibrin has numerous binding sites for not only cells, but for growth factors and other ECM components as well. Fibrin sequesters a multitude of growth factors which are essential for bone regeneration including FGF, VEGF, PDGF, IGF, and the TGF-β superfamily including BMP-2 (Martino and Hubbell, 2010). Thus, fibrin ultimately also provides a repository of signaling cues to direct cell behavior (Brown and Barker, 2014).

Fibrin is degraded (via fibrinolysis) into nontoxic components by the serine protease plasmin, which is initiated by the coagulation cascade. The rate of degradation depends on the fibrin fiber thickness and density; a fibrin network comprised of dense thin fibers degrades much more slowly than a network of thick loose fibers (Weisel, 2007). Fiber thickness has also been shown to play a large role in cell-specific signaling molecule expression (Shats et al., 1997). Fibrin material properties can be altered through polymerization by adjusting the concentrations of fibrinogen, thrombin, and Ca^2+^/salt concentrations (Cox et al., 2004). In general, increasing fibrinogen concentration leads to increasingly dense plug formation. However, care must be taken not to make the hydrogel too dense or else it will inhibit cell migration (Karp et al., 2004). Decreasing thrombin concentration leads to increased material modulus, ultimate tensile strength, and increasing fibrin fiber diameters (Rowe et al., 2007). Sodium chloride concentration has an effect on hydrogel compressive modulus and fiber diameter, which in turn affects MSC differentiation and alkaline phosphatase expression (Davis et al., 2011). With all of these factors in mind, fibrin biomaterials have been used for decades for cell and biomolecule delivery (Rajangam and An, 2013).

Fibrin is FDA approved as a wound sealant, and clinically, fibrin sealants have been mixed with hydroxyapatite for craniofacial applications (Le Guehennec et al., 2004). Isoelectric points of fibrinogen (pH 5.5) and fibrin (pH 5.6) (Oka, 1981) would cause fibrin to have negative surface charge at the neutral pH, which would allow for rhBMP-2 “entrapment” and adsorption of nucleic acid complexes (Saul et al., 2007).

#### Keratin

Keratins are intermediate filament proteins and intermediate filament-associated proteins, which are widely found in nature and are best known as being the structural proteins referred to as hard or soft keratins. Wool and human hair are commonly used as sources for biomaterials due to their availability, renewable source, low toxicity, ease of sterilization, and ability to be hydrolytically degraded. Keratins can be processed into a number of biomaterials such as electrospun fibers, (Xing et al., 2010), scaffolds (Tachibana et al., 2002; Srinivasan et al., 2010), and films (Yamauchi et al., 2003). One salient feature of keratins is the high number of cysteine residues, which can form disulfide bonds. This allows for control over the number of disulfide crosslinks within the material and thus the material properties and rates of degradation (Crewther et al., 1965). One approach is the chemical modification of cysteine residues via alkylation on keratin extracted by reductive means (known as kerateine) (Tachibana et al., 2006; Han et al., 2015). Alternatively, different chemical forms of the keratin resulting from oxidative (keratose) or reductive (kerateine) extraction can be mixed to tune the properties of the materials in a similar fashion to alkylation (Ham et al., 2015).

Keratin can be used as a traditional sponge (Tachibana et al., 2002; Katoh et al., 2004) or injectable gel (Aboushwareb et al., 2009; Saul et al., 2011; Kowalczewski et al., 2014) which allows for minimally invasive administration able to contour to any defect shape. A number of studies have already shown keratin's ability to achieve healing in injured bone as a carrier for hydroxyapatite (Tachibana et al., 2005; Dias et al., 2010a,b). Other studies have demonstrated healing in long bone (de Guzman et al., 2013) and mandible (Kowalczewski et al., 2014) models when keratin is used as a carrier for rhBMP-2.

#### Silk

Silks are proteins well-known for the high number of beta sheets in their secondary structure. Silk contains integrin binding motifs and is an appealing biomaterial for bone applications due to its very high tensile strength, osteoconductive nature (Meinel et al., 2005), and predictable rate of degradation (Altman et al., 2003). The two primary sources of silk are spider or silkworm (Arntzen and Ritter, 1994), and these can also be modified (e.g., by crosslinking tyrosine residues) to provide a more tunable carrier platform (Partlow et al., 2014). Silks can also be processed into a number of forms including films (Dutta et al., 2013), fibers (Mandal et al., 2012; Panda et al., 2015), hydrogels (McGill et al., 2017), scaffolds (Marolt et al., 2006; Zhu et al., 2014), and are compatible for fabrication of composites with ceramic (Jin et al., 2015) or synthetic polymers. Furthermore, these materials have been used as carriers for rhBMP-2 (Karageorgiou et al., 2006; Ma et al., 2016) and promoted bridging in a critically-sized rat femur defect model (Kirker-Head et al., 2007). Interestingly, domestication of silkworms (i.e., diet and environmental conditions) has resulted in higher silk yields compared to wild silkworms (Normile, 2009); however, it has resulted in alterations in silk properties (Holland et al., 2012; Fang et al., 2015). In particular, silk from silkworms fed non-mulberry diet have shown to be superior in applications for bone regeneration (Naskar et al., 2014; Sahu et al., 2015; Midha et al., 2016).

#### Alginate, chitosan, and other polysaccharides

Each of the materials noted above (keratin, fibrin, silk) are protein-based materials. Polysaccharide-based materials have also been investigated as a potential carrier system. One drawback to many polysaccharides is that they do not bear the amino acid sequences typically associated with cell attachment via integrin binding domains. Cell attachment and osteoconductivity in polysaccharides are often enhanced by methods that include mixing with osteoconductive materials such as hydroxyapatite (Danilchenko et al., 2011), chemical surface modifications (Luna et al., 2011), or incorporating cell adhesion proteins such as collagen (Lawson et al., 2004) or fibronectin (Kirchhof and Groth, 2008) or integrin binding sequences such as arginine-glycine-aspartic acid (RGD) (Ho et al., 2017). Although peripheral to this review, it is noteworthy that alginate has long been used for cell encapsulation techniques (e.g., for xenogeneic or allogeneic islet cell implantation for production of insulin for Type I diabetes; Lim and Sun, 1980). The ability to encapsulate cells and control matrix stiffness to mediate (stem) cell response makes such materials particularly interesting for cell-based approaches to bone repair (Darnell et al., 2017). A potential advantage of polysaccharide materials, when used alone or as part of a composite material, is their suitability for controlled release of small molecule drugs (Nafee et al., 2017), growth factors (Cao et al., 2017), and nucleic acids (Li et al., 2017) with or without cells.

## Molecular targets to promote bone regeneration

### Molecular promoters of bone regeneration

#### TGF-β superfamily and BMPs

In considering potential therapeutics to promote bone regeneration, it is informative to consider the molecular nature of the bone healing process. Molecular cues expressed during healing can be divided into three broad categories: (1) pro-inflammatory cytokines, (2) angiogenic factors, and (3) the transforming growth factor beta (TGF-β) superfamily and other growth factors (Ai-Aql et al., 2008).

Of the endogenous growth factors involved in bone healing, possibly none are as important as the transforming growth factor-beta (TGF-β) superfamily which acts upon a broad range of cells, influencing cellular activity, growth, differentiation, and extracellular matrix production. After initial blood clot formation, platelets release TGF-β to stimulate the proliferation of periosteal cells. Even though TGF-β plays a role in the production of extracellular proteins for callus formation, its osteoinductive potential is limited and does not have an effect on mineralization (Lind et al., 1993). TGF-β's most important roles may lay in initiating the production of BMPs in osteoprogenitor cells while inhibiting osteoclast activation (Dimitriou et al., 2005).

The most important members of the TGF-β superfamily, in terms of bone regeneration, are likely the BMPs. BMPs are pleiotropic regulators of growth (Friedrichs et al., 2011), differentiation (Pera et al., 2004), and apoptosis (Hyzy et al., 2012) of a variety of cell types. One property of BMPs is their ability to be osteoinductive by themselves (especially BMP-2,-6,-7, and -9; Termaat et al., 2005), which makes them attractive therapeutically for tissue engineering products. They are strong promoters of differentiation of osteoprogenitor cells into osteoblasts. BMPs are expressed at various points along the phases of bone healing. BMP-2, -6, and -9 have been found to be the most potent inducers of pluripotent MSCs to differentiate into osteoblasts. Most other BMPs act more to support the terminal differentiation and maturation of osteocytes (Cheng et al., 2003). As previously discussed, to-date only BMP-2 and BMP-7 have been approved for clinical use in the United States for bone substitutes. While BMP-2, in particular, has achieved clinical and commercial success, its side effects due to supraphysiological dosage suggest that other molecular targets might be used to reduce the dosages required to achieve bone healing.

One approach to this end is the use of peptide sequences or low molecular weight drugs sequences. Peptide sequences which consist of small components of BMP-2, can used to promote bone healing while minimizing negative side effects (Saito et al., 2004; Li et al., 2011). The concept here is to identify the components responsible for promoting bone healing while minimizing negative side effects. Another approach is to use small molecules to facilitate the action of BMP-2 at lower doses. For example, the SVAK-12 compound interacts with the Smad binding site of Smurf-1 to prevent degradation of Smad, which play a role in the BMP/TGF-β signaling pathway (Kato et al., 2011). Other drugs such as simvastatin (Qi et al., 2013) and lovastatin (Yoshii et al., 2014) also work on the BMP/Smad pathway while bisphosphonates (Stadelmann et al., 2008), for example, work on alternative pathways. A particularly intriguing approach is the modification of known growth factors with so-called superaffinity domains, which allows these growth factors to achieve effects at a lower effective dose through better binding affinity to their carrier material or ECM proteins (Martino et al., 2014).

While each of the above approaches has advantages, there are several continuing challenges. One is that the potency of these molecules seems to be lower than BMP-2 with, for example, the P24 peptide having a dose three orders of magnitude greater than rhBMP-2 (Wu et al., 2008; Li et al., 2011), and thus requiring a larger overall mass to be delivered. Secondly, due to their smaller size, these molecules are susceptible to more rapid diffusion from their carriers, potentially shortening their activity.

#### Angiogenesis

An issue that has long been known but is yet to be fully solved for tissue engineering strategies is the need for vascularization of newly formed tissue (Nerem, 2006). In injured bone the initial hypoxic conditions during fracture healing stimulate angiogenesis, which is crucial for successful bone healing (Rowe et al., 1999). Platelet-derived growth factor (PDGF) is released from platelets entrapped within the hematoma in the early stages of bone healing and up-regulates vascular endothelial growth factor (VEGF) (Hankenson et al., 2011). The expression of VEGF (Deckers et al., 2002) and PDGF (Xie et al., 2014) by pre-osteogenic cells has been shown to be a crucial component in regulating the rate of neo-angiogenesis to correlate with the rate of bone formation (Gerstenfeld et al., 2003). In addition PDGF acts as a potent mitogen of inflammatory cells and mesenchymal stem cells (MSCs) (Dimitriou et al., 2005). Vascular ingrowth is also regulated by fibroblast growth factor (FGF) secreted by macrophages, mesenchymal stem cells, chondrocytes, and osteoblasts. FGF-1 and FGF-2, being the most commonly expressed growth factors in bone regeneration, partake by increasing callus formation and osteoblast activity (Nakamura et al., 1998). Although angiogenic factors are crucial for effective bone healing, alone they are not osteoinductive. It might be expected that the combination of angio-genic factors in combination with BMP-2 might promote formation of more rapid healing or higher quality bone. However, combinatorial approaches have yielded minimal improvements (Patel et al., 2008), highlighting the need for highly tuned temporal control of delivery in such strategies (Young et al., 2009).

#### Cytokines and other growth factors

Pro-inflammatory cytokines interleukin-1 (IL-1), interleukin6 (IL-6), and tumor necrosis factor alpha (TNF-α) are secreted within the first 24 h of bone damage by macrophages which causes the initiation of the repair phase of bone regeneration. These cytokines initiate the downstream responses that, in-turn, initiate the induction of both angiogenic factors and growth factors. TNF-α activates osteoclast activity for the removal of bone debris and promotes recruitment of mesenchymal stem cells. The expression of these pro-inflammatory cytokines is highest during the first 24 h of bone healing and the cytokines are subsequently expressed in smaller quantities during repair and remodeling phase (Kalfas, 2001). The ability to direct inflammatory response is therefore a potentially potent mediator of bone healing.

Insulin-like growth factors (IGF) play critical roles in skeletal development as well as fracture healing by promoting bone matrix formation such as collagen type 1 (Tsiridis et al., 2007). IGF-1 is the most potent in the IGF family and is localized in healing fractures (Andrew et al., 1993). IGF-1 stimulates chemotaxis and activity of osteoblasts, and has the greatest effect on bone formation when it is used in combination with TGF-β (Schmidmaier et al., 2003). One advantage of BMP-2 strategies is that this molecule is sufficient and necessary in the natural regeneration process, which has allowed it to be used alone to promote bone regeneration. Strategies that employ other and multiple growth factors may require improved understanding of the underlying biology and strategies to precisely time the delivery of these molecules. Although beyond the focus of this review, the advancement of small molecules and osteogenic drugs in bone regeneration can serve as alternatives to exogenous growth factors and cytokines (Han et al., 2013; Laurencin et al., 2014; Balmayor, 2015). A number of small molecules have been used alone (Papadimitriou et al., 2015) or in combination with growth factors such as rhBMP-2 (Cho et al., 2017) with success in bone regeneration. However, due to non-specific cellular uptake, adverse effects due to unwanted signaling cascades, and lack of efficient local sustained delivery (Brouwers et al., 2011; Laurencin et al., 2014) are limiting factors in the current progress of small-molecules.

### Molecular inhibitors of bone regeneration

Molecular control over fracture healing follows developmental osteogenesis very closely (Gilbert, 2000). Patterning and maintenance of tissue is overseen not only through molecular promoters, such as those discussed above, but is also by molecular inhibitors. Recent interest, as noted above for the SVAK-12 compound, has focused on bone regeneration inhibitors in particular due to their mechanistic role in which negative feedback and crosstalk decrease the cellular exposure of the molecular promoters of bone regeneration. The effectiveness of these signaling regulators and how they can have significant negative impact on BMP efficacy can be appreciated when considering the therapeutic dose of BMPs in bone substitutes. One reason that BMP carriers are loaded with supraphysiological concentrations is likely related to the need to overcome the regulating factors of BMP inhibitors in order to achieve a therapeutic response. These inhibitors are present within the BMP signaling cascade at (1) intracellular locations, (2) as pseudo-receptors, and (3) in extracellular locations.

Intracellular inhibitors of BMP signaling include inhibitory SMADs which are dormant in the nucleus until BMP stimulation at which time they are released into the cytoplasm. After BMP binds to its receptor, SMADs inhibit the signal transduction by interacting with the BMP receptor. SMAD-6 specifically acts to inhibit BMP signaling while SMAD-7 targets the general TGF-β superfamily (Ishisaki et al., 1999). Another intracellular regulator is SMAD ubiquitin regulatory factor (SMURF), which controls intracellular BMP signal transduction by binding and degrading various positive signaling molecules or by degrading the BMP receptor (Murakami et al., 2003).

BMP and activin membrane bound inhibitor (BAMBI) is a pseudo-receptor which presents an extracellular domain similar to a BMP receptor domain; the difference is that BAMBI lacks the intracellular domain. Therefore, when BMPs bind to a pseudo-receptor it cannot form an active receptor complex in order to propagate the signal (Tsiridis et al., 2007).

Extracellular inhibitors, mainly produced by osteoblasts, are secreted proteins that act as BMP binding antagonists which prevent BMP from binding with its receptors. An increase in extracellular inhibitor expression is directly correlated with an increase in local BMP levels. The majority of the binding antagonists focus on BMPs with the strongest osteoinductive potential (BMP-2,-6, and -9; Termaat et al., 2005). Even though BMPs are mainly associated with being molecular promoters of bone regeneration, BMP-3 is an antagonist of osteogenic BMPs. Ironically, it is the most abundantly expressed BMP in adult bone (Tsiridis et al., 2007). The differential screening-selected gene aberrative in neuroblastoma (DAN) family proteins include gremlin and sclerostin. Gremlin binds and blocks BMP-2,-4, and -7. Gremlin is responsible for inhibiting osteoblast differentiation and reducing bone remodeling (Dimitriou et al., 2006). Sclerostin is produced by osteoclasts and directly competes with BMP-2,-4,-6, and -7 in binding to their receptors to inhibit osteoblast differentiation and bone remodeling. Sclerostin not only decreases MSC differentiation and osteoblast activity but also induces apoptosis in bone cells. Follistatin neutralizes BMP-2,-4,-15, and joins with high affinity to BMP-7 by forming a trimeric complex between itself, BMP, and the receptor (Abe et al., 2004). In embryogenesis, follistatin is known to inhibit all aspects of BMP activity (Iemura et al., 1998), but its role in adult bone healing is yet to be completely understood. Chordin binds BMP-2,-4, and -7 and blocks their ability to bind to BMP receptors and acts similarly to gremlin (Canalis et al., 2003). Noggin has the ability to bind to the greatest number of molecular promoters of bone regeneration: BMP-2,-4,-5,-6, and -7 and prevents them from binding to BMP receptors. Noggin works in a complementary fashion with gremlin to develop a local zone which is devoid of BMP (Stafford et al., 2011).

#### Interfering RNA approaches to removing inhibitors/antagonists

Although each of the molecules discussed above play inhibitory or antagonistic roles in response to BMPs, they also play important roles in the homeostasis of healthy bone. As such, approaches to temporarily (not permanently) remove these molecules are important.

Small interfering RNA (siRNA) is a gene-silencing mechanism by which post-transcriptional gene silencing can occur (Cheema et al., 2007). Delivery of siRNA alone is not successful due to its susceptibility to degradation and overall negative charge, which prevents siRNA from passing through the cell membrane (Wang J. et al., 2010). To overcome these challenges and allow siRNA to be effective in the cytoplasm of targeted cells, siRNA can be packaged into viral or non-viral vectors.

Of particular interest in tissue engineering approaches is the ability to easily incorporate these constructs into biomaterial scaffolds. Krebs et al. were among the first to demonstrate the ability of a three dimensional hydrogel to achieve delivery of siRNA in a sustainable fashion (Krebs et al., 2009). Local delivery strategies now involve layer-by-layer (Hossfeld et al., 2013), nanoparticle embedding (Mittnacht et al., 2010), and direct incorporation into a cationic hydrogel (Ma et al., 2014).

In terms of promoting bone regeneration, focus has been on co-delivery of inductive molecules such as BMP-2 and siRNA targeting suppressive molecules. Targeting intracellular regulators of BMP-2, such as Smurf1 (Rodriguez-Evora et al., 2014), resulted in improved osteogenic activity of MSCs. “Pre-treatment” with noggin siRNA has also led to significantly increased expression of osteogenic markers in *in vitro*, and of bone regeneration *in vivo* (Wan et al., 2007). Substrate-mediated siRNA delivery for bone application has just begun to make an impact on the scientific community. The use of chitosan hydrogels as a reservoir for siRNA delivery has shown successful down regulation of osteoclast activity *in vitro* (Ma et al., 2014). Substrate-mediated delivery of noggin siRNA from a synthetic polymer has successfully enhanced osteogenic activity *in vitro* (Nguyen et al., 2014) and we recently achieved delivery of noggin siRNA from the surface of fibrin hydrogel films (Kowalczewski and Saul, 2015).

## Conclusions

In order to address the challenges and drawbacks of current augmentation strategies for critically-sized bone defects, tissue engineered bone substitutes have been designed to be both osteoconductive (collagen carrier) and osteoinductive (rhBMP-2). Like many first-generation products, there have been a number of drawbacks to rhBMP-2 collagen carriers such as edema and ectopic bone growth. In order to develop the next generation of bone substitutes it is important to understand the biological action and temporal expression during the healing cascade. Currently the focus has been placed on only incorporating molecules which promote bone regeneration (BMPs) without acknowledging the innate molecular controls achieved with inhibitory molecules (e.g., Noggin, Gremlin). In order to successfully decrease the therapeutic concentration of BMPs, novel carrier systems that maintain or enhance rhBMP-2 bioactivity must be designed and the negative feedback signaling caused by BMP antagonists must be addressed.

## Author contributions

CK conceived of topics in the review, wrote initial draft of most sections of the manuscript, and conducted editing of the document; JS conceived of concept for the review for article, wrote initial draft of several sections of the manuscript, provided final revisions, and primary editing of the document.

### Conflict of interest statement

One of the authors is a former employee of KeraNetics, LLC (CK) and both authors (CK and JS) have received grant funding through KeraNetics, LLC within the past 5 years related to keratin research. One of the authors (JS) has a patent pending related to keratin biomaterials for drug delivery and 2 patents related to cell encapsulation strategies with alginate. The reviewer JX and handling Editor declared their shared affiliation.

## References

[B1] Abbah S. A. Liu J. Goh J. C. Wong H. K. (2013). Enhanced control of *in vivo* bone formation with surface functionalized alginate microbeads incorporating heparin and human bone morphogenetic protein-2. Tissue Eng. Part A 19, 350–359. 10.1089/ten.tea.2012.0274 22894570 PMC3542875

[B2] Abe Y. Abe T. Aida Y. Hara Y. Maeda K. (2004). Follistatin restricts bone morphogenetic protein (BMP)-2 action on the differentiation of osteoblasts in fetal rat mandibular cells. J. Bone Miner. Res. 19, 1302–1307. 10.1359/JBMR.040408 15231018

[B3] Aboushwareb T. Eberli D. Ward C. Broda C. Holcomb J. Atala A.. (2009). A keratin biomaterial gel hemostat derived from human hair: evaluation in a rabbit model of lethal liver injury. J. Biomed. Mater. Res. Part B Appl. Biomater. 90, 45–54. 10.1002/jbm.b.31251 18988274

[B4] Ai-Aql Z. S. Alagl A. S. Graves D. T. Gerstenfeld L. C. Einhorn T. A. (2008). Molecular mechanisms controlling bone formation during fracture healing and distraction osteogenesis. J. Dent. Res. 87, 107–118. 10.1177/154405910808700215 18218835 PMC3109437

[B5] Aksakal B. Demirel M. (2017). Synthesis and characterization of Bioglass-based bone grafts with Gelatine substitution for biomedical applications. Biomed. Mater. Eng. 28, 159–168. 10.3233/BME-171664 28372268

[B6] Altman G. H. Diaz F. Jakuba C. Calabro T. Horan R. L. Chen J.. (2003). Silk-based biomaterials. Biomaterials 24, 401–416. 10.1016/S0142-9612(02)00353-8 12423595

[B7] Alvear J. Artaza C. Vial M. Guerrero S. Muzzo S. (1986). Physical growth and bone age of survivors of protein energy malnutrition. Arch. Dis. Child. 61, 257–262. 10.1136/adc.61.3.257 3083790 PMC1777696

[B8] Andrew J. G. Hoyland J. Freemont A. J. Marsh D. (1993). Insulinlike growth factor gene expression in human fracture callus. Calcif. Tissue Int. 53, 97–102. 10.1007/BF01321886 8402329

[B9] Arntzen C. J. Ritter E. M. (1994). Encyclopedia of Agricultural Science. San Diego, CA: Academic Press.

[B10] Ashley G. W. Henise J. Reid R. Santi D. V. (2013). Hydrogel drug delivery system with predictable and tunable drug release and degradation rates. Proc. Natl. Acad. Sci. U.S.A. 110, 2318–2323. 10.1073/pnas.1215498110 23345437 PMC3568318

[B11] Athanasiou K. A. Niederauer G. G. Agrawal C. M. (1996). Sterilization, toxicity, biocompatibility and clinical applications of polylactic acid/polyglycolic acid copolymers. Biomaterials 17, 93–102. 10.1016/0142-9612(96)85754-1 8624401

[B12] Bae H. Zhao L. Zhu D. Kanim L. E. Wang J. C. Delamarter R. B. (2010). Variability across ten production lots of a single demineralized bone matrix product. J. Bone Joint Surg. Am. 92, 427–435. 10.2106/JBJS.H.01400 20124070

[B13] Bae S. E. Choi J. Joung Y. K. Park K. Han D. K. (2012). Controlled release of bone morphogenetic protein (BMP)-2 from nanocomplex incorporated on hydroxyapatite-formed titanium surface. J. Control. Release 160, 676–684. 10.1016/j.jconrel.2012.04.021 22543042

[B14] Balmayor E. R. (2015). Targeted delivery as key for the success of small osteoinductive molecules. Adv. Drug Deliv. Rev. 94, 13–27. 10.1016/j.addr.2015.04.022 25959428

[B15] Barbieri D. Yuan H. Ismailoglu A. S. de Bruijn J. D. (2017). Comparison of two moldable calcium phosphate-based bone graft materials in a noninstrumented canine interspinous implantation model. Tissue Eng. Part A 23, 1310–1320. 10.1089/ten.tea.2016.0347 28132596

[B16] Barrett K. E. (2010). Ganong's Review of Medical Physiology. New York, NY: McGraw-Hill Medical.

[B17] Bishop G. B. Einhorn T. A. (2007). Current and future clinical applications of bone morphogenetic proteins in orthopaedic trauma surgery. Int. Orthop. 31, 721–727. 10.1007/s00264-007-0424-8 17668207 PMC2266667

[B18] BMUS (2014). United States Bone and Joint Initiative: The Burden of Musculoskeletal Diseases in the United States (BMUS), 3rd Edn Rosemont, IL Available online at: http://www.boneandjointburden.org (Accessed July 29, 2014).

[B19] Bose S. Roy M. Bandyopadhyay A. (2012). Recent advances in bone tissue engineering scaffolds. Trends Biotechnol. 30, 546–554. 10.1016/j.tibtech.2012.07.005 22939815 PMC3448860

[B20] Brouwers L. Iskar M. Zeller G. van Noort V. Bork P. (2011). Network neighbors of drug targets contribute to drug side-effect similarity. PLoS ONE 6:e22187. 10.1371/journal.pone.0022187 21765950 PMC3135612

[B21] Brown A. C. Barker T. H. (2014). Fibrin-based biomaterials: modulation of macroscopic properties through rational design at the molecular level. Acta Biomater. 10, 1502–1514. 10.1016/j.actbio.2013.09.008 24056097 PMC3960324

[B22] Brown K. V. Li B. Guda T. Perrien D. S. Guelcher S. A. Wenke J. C. (2011). Improving bone formation in a rat femur segmental defect by controlling bone morphogenetic protein-2 release. Tissue Eng. Part A 17, 1735–1746. 10.1089/ten.tea.2010.0446 21338268

[B23] Canalis E. Economides A. N. Gazzerro E. (2003). Bone morphogenetic proteins, their antagonists, and the skeleton. Endocr. Rev. 24, 218–235. 10.1210/er.2002-0023 12700180

[B24] Cao L. Werkmeister J. A. Wang J. Glattauer V. McLean K. M. Liu C. (2014). Bone regeneration using photocrosslinked hydrogel incorporating rhBMP-2 loaded 2-N, 6-O-sulfated chitosan nanoparticles. Biomaterials 35, 2730–2742. 10.1016/j.biomaterials.2013.12.028 24438908

[B25] Cao L. Yu Y. Wang J. Werkmeister J. A. McLean K. M. Liu C. (2017). 2-N, 6-O-sulfated chitosan-assisted BMP-2 immobilization of PCL scaffolds for enhanced osteoinduction. Mater. Sci. Eng. C 74, 298–306. 10.1016/j.msec.2016.12.004 28254298

[B26] Cheema S. K. Chen E. Shea L. D. Mathur A. B. (2007). Regulation and guidance of cell behavior for tissue regeneration via the siRNA mechanism. Wound Repair Regen. 15, 286–295. 10.1111/j.1524-475X.2007.00228.x 17537114

[B27] Cheng H. Jiang W. Phillips F. M. Haydon R. C. Peng Y. Zhou L. (2003). Osteogenic activity of the fourteen types of human bone morphogenetic proteins (BMPs). J. Bone Joint Surg. Am. 85-A, 1544–1552. 10.2106/00004623-200308000-00017 12925636

[B28] Chiou B. S. English R. J. Khan S. A. (1996). Rheology and photo-cross-linking of thiol-ene polymers. Macromolecules 29, 5368–5374. 10.1021/ma960383e

[B29] Cho T. H. Kim I. S. Lee B. Park S. N. Ko J. H. Hwang S. J. (2017). (^*^) Early and marked enhancement of new bone quality by alendronate-loaded collagen sponge combined with bone morphogenetic protein-2 at high dose: a long-term study in calvarial defects in a rat model. Tissue Eng. Part A 23, 1343–1360. 10.1089/ten.tea.2016.0557 28457207

[B30] Clark R. A. Lanigan J. M. DellaPelle P. Manseau E. Dvorak H. F. Colvin R. B. (1982). Fibronectin and fibrin provide a provisional matrix for epidermal cell migration during wound reepithelialization. J. Invest. Dermatol. 79, 264–269. 10.1111/1523-1747.ep12500075 6752288

[B31] Cox S. Cole M. Tawil B. (2004). Behavior of human dermal fibroblasts in three-dimensional fibrin clots: dependence on fibrinogen and thrombin concentration. Tissue Eng. 10, 942–954. 10.1089/1076327041348392 15265312

[B32] Crewther W. G. Fraser R. D. Lennox F. G. Lindley H. (1965). The chemistry of keratins. Adv. Protein Chem. 20, 191–346. 10.1016/S0065-3233(08)60390-3 5334826

[B33] Cutter C. S. Mehrara B. J. (2006). Bone grafts and substitutes. J. Long Term Eff. Med. Implants 16, 249–260. 10.1615/JLongTermEffMedImplants.v16.i3.50 17073567

[B34] Daley W. P. Peters S. B. Larsen M. (2008). Extracellular matrix dynamics in development and regenerative medicine. J. Cell Sci. 121, 255–264. 10.1242/jcs.006064 18216330

[B35] Danilchenko S. N. Kalinkevich O. V. Pogorelov M. V. Kalinkevich A. N. Sklyar A. M. Kalinichenko T. G.. (2011). Characterization and in vivo evaluation of chitosan-hydroxyapatite bone scaffolds made by one step coprecipitation method. J. Biomed. Mater. Res. A 96, 639–647. 10.1002/jbm.a.33017 21268238

[B36] Darnell M. Young S. Gu L. Shah N. Lippens E. Weaver J. (2017). Substrate stress-relaxation regulates scaffold remodeling and bone formation *in vivo*. Adv. Healthc. Mater. 6:1601185 10.1002/adhm.201601185 PMC5440842 27995768

[B37] Davis H. E. Miller S. L. Case E. M. Leach J. K. (2011). Supplementation of fibrin gels with sodium chloride enhances physical properties and ensuing osteogenic response. Acta Biomater. 7, 691–699. 10.1016/j.actbio.2010.09.007 20837168

[B38] Deckers M. M. van Bezooijen R. L. van der Horst G. Hoogendam J. van Der Bent C. Papapoulos S. E.. (2002). Bone morphogenetic proteins stimulate angiogenesis through osteoblast-derived vascular endothelial growth factor A. Endocrinology 143, 1545–1553. 10.1210/endo.143.4.8719 11897714

[B39] de Guzman R. C. Saul J. M. Ellenburg M. D. Merrill M. R. Coan H. B. Smith T. L.. (2013). Bone regeneration with BMP-2 delivered from keratose scaffolds. Biomaterials 34, 1644–1656. 10.1016/j.biomaterials.2012.11.002 23211447

[B40] Del Rosario C. Rodriguez-Evora M. Reyes R. Simoes S. Concheiro A. Evora C.. (2015). Bone critical defect repair with poloxamine-cyclodextrin supramolecular gels. Int. J. Pharm. 495, 463–473. 10.1016/j.ijpharm.2015.09.003 26362078

[B41] Dias G. J. Mahoney P. Swain M. Kelly R. J. Smith R. A. Ali M. A. (2010a). Keratin-hydroxyapatite composites: biocompatibility, osseointegration, and physical properties in an ovine model. J. Biomed. Mater. Res. A 95, 1084–1095. 10.1002/jbm.a.32908 20878901

[B42] Dias G. J. Peplow P. V. McLaughlin A. Teixeira F. Kelly R. J. (2010b). Biocompatibility and osseointegration of reconstituted keratin in an ovine model. J. Biomed. Mater. Res. A 92, 513–520. 10.1002/jbm.a.32394 19213058

[B43] Di Luca A. Ostrowska B. Lorenzo-Moldero I. Lepedda A. Swieszkowski W. Van Blitterswijk C.. (2016). Gradients in pore size enhance the osteogenic differentiation of human mesenchymal stromal cells in three-dimensional scaffolds. Sci. Rep. 6:22898. 10.1038/srep22898 26961859 PMC4790631

[B44] Dimitriou R. Tsiridis E. Carr I. Simpson H. Giannoudis P. V. (2006). The role of inhibitory molecules in fracture healing. Injury 37(Suppl. 1), S20–S29. 10.1016/j.injury.2006.02.039 16616754

[B45] Dimitriou R. Tsiridis E. Giannoudis P. V. (2005). Current concepts of molecular aspects of bone healing. Injury 36, 1392–1404. 10.1016/j.injury.2005.07.019 16102764

[B46] Dutta S. Talukdar B. Bharali R. Rajkhowa R. Devi D. (2013). Fabrication and characterization of biomaterial film from gland silk of muga and eri silkworms. Biopolymers 99, 326–333. 10.1002/bip.22168 23426575

[B47] Ebara S. Nakayama K. (2002). Mechanism for the action of bone morphogenetic proteins and regulation of their activity. Spine 27, S10–S15. 10.1097/00007632-200208151-00004 12205413

[B48] Efstathopoulos N. Giamarellos-Bourboulis E. Kanellakopoulou K. Lazarettos I. Giannoudis P. Frangia K.. (2008). Treatment of experimental osteomyelitis by methicillin resistant *Staphylococcus aureus* with bone cement system releasing grepafloxacin. Injury 39, 1384–1390. 10.1016/j.injury.2008.04.006 18656187

[B49] Fang S. M. Hu B. L. Zhou Q. Z. Yu Q. Y. Zhang Z. (2015). Comparative analysis of the silk gland transcriptomes between the domestic and wild silkworms. BMC Genomics 16:60 10.1186/s12864-015-1287-9 25887670 PMC4328555

[B50] Feng X. Tonnesen M. G. Mousa S. A. Clark R. A. (2013). Fibrin and collagen differentially but synergistically regulate sprout angiogenesis of human dermal microvascular endothelial cells in 3-dimensional matrix. Int. J. Cell Biol. 2013:231279 10.1155/2013/231279 23737792 PMC3657431

[B51] Freeman M. A. Bradley G. W. Revell P. A. (1982). Observations upon the interface between bone and polymethylmethacrylate cement. J. Bone Joint Surg. Br. 64, 489–493. 10.1302/0301-620X.64B4.7096429 7096429

[B52] Friedrichs M. Wirsdoerfer F. Flohe S. B. Schneider S. Wuelling M. Vortkamp A. (2011). BMP signaling balances proliferation and differentiation of muscle satellite cell descendants. BMC Cell Biol. 12:26 10.1186/1471-2121-12-26 21645366 PMC3149017

[B53] Friess W. Uludag H. Foskett S. Biron R. Sargeant C. (1999). Characterization of absorbable collagen sponges as rhBMP-2 carriers. Int. J. Pharm. 187, 91–99. 10.1016/S0378-5173(99)00174-X 10502616

[B54] Gerstenfeld L. C. Cullinane D. M. Barnes G. L. Graves D. T. Einhorn T. A. (2003). Fracture healing as a post-natal developmental process: molecular, spatial, and temporal aspects of its regulation. J. Cell. Biochem. 88, 873–884. 10.1002/jcb.10435 12616527

[B55] Gilbert S. F. (ed.). (2000). Osteogenesis: the development of bones, in Developmental Biology, 6th Edn. (Sunderland, MA: Sinauer Associates), 454–458.

[B56] Greenwald A. S. Boden S. D. Goldberg V. M. Khan Y. Laurencin C. T. Rosier R. N. (2001). The Committee on Biological, Bone-graft substitutes: facts, fictions, and applications. J. Bone Joint Surg. Am. 83-A(Suppl. 2) (Pt 2), 98–103. 10.2106/00004623-200100022-00007 11712842

[B57] Grover V. Kapoor A. Malhotra R. Sachdeva S. (2011). Bone allografts: a review of safety and efficacy. Indian J. Dent. Res. 22, 496. 10.4103/0970-9290.87084 22048602

[B58] Gruskin E. Doll B. A. Futrell F. W. Schmitz J. P. Hollinger J. O. (2012). Demineralized bone matrix in bone repair: history and use. Adv. Drug Deliv. Rev. 64, 1063–1077. 10.1016/j.addr.2012.06.008 22728914 PMC7103314

[B59] Guda T. Walker J. A. Singleton B. Hernandez J. Oh D. S. Appleford M. R. (2014). Hydroxyapatite scaffold pore architecture effects in large bone defects *in vivo*. J. Biomater. Appl. 28, 1016–1027. 10.1177/0885328213491790 23771772

[B60] Ham T. R. Lee R. T. Han S. Haque S. Vodovotz Y. Gu J.. (2015). Tunable keratin hydrogels for controlled erosion and growth factor delivery. Biomacromolecules 17, 225–236. 10.1021/acs.biomac.5b01328 26636618 PMC5565161

[B61] Han Q. Q. Du Y. Yang P. S. (2013). The role of small molecules in bone regeneration. Future Med. Chem. 5, 1671–1684. 10.4155/fmc.13.133 24047272

[B62] Han S. Ham T. R. Haque S. Sparks J. L. Saul J. M. (2015). Alkylation of human hair keratin for tunable hydrogel erosion and drug delivery in tissue engineering applications. Acta Biomater. 23, 201–213. 10.1016/j.actbio.2015.05.013 25997587 PMC4522204

[B63] Hankenson K. D. Dishowitz M. Gray C. Schenker M. (2011). Angiogenesis in bone regeneration. Injury 42, 556–561. 10.1016/j.injury.2011.03.035 21489534 PMC3105195

[B64] Harwood P. J. Newman J. B. Michael L. R. (2010). (ii) An update on fracture healing and non-union. Orthop. Trauma 24, 9–23. 10.1016/j.mporth.2009.12.004

[B65] Hernandez A. Sanchez E. Soriano I. Reyes R. Delgado A. Evora C. (2012). Material-related effects of BMP-2 delivery systems on bone regeneration. Acta Biomater. 8, 781–791. 10.1016/j.actbio.2011.10.008 22023753

[B66] Hersel U. Dahmen C. Kessler H. (2003). RGD modified polymers: biomaterials for stimulated cell adhesion and beyond. Biomaterials 24, 4385–4415. 10.1016/S0142-9612(03)00343-0 12922151

[B67] Ho S. S. Keown A. T. Addison B. Leach J. K. (2017). Cell migration and bone formation from mesenchymal stem cell spheroids in alginate hydrogels are regulated by adhesive ligand density. Biomacromolecules 18, 4331–4340. 10.1021/acs.biomac.7b01366 29131587 PMC5971090

[B68] Holland C. Porter D. Vollrath F. (2012). Comparing the rheology of mulberry and “wild” silkworm spinning dopes. Biopolymers 97, 362–367. 10.1002/bip.22011 22161905

[B69] Hollister S. J. Flanagan C. L. Zopf D. A. Morrison R. J. Nasser H. Patel J. J.. (2015). Design control for clinical translation of 3D printed modular scaffolds. Ann. Biomed. Eng. 43, 774–786. 10.1007/s10439-015-1270-2 25666115 PMC4407657

[B70] Hollister S. J. Lin C. Y. Saito E. Schek R. D. Taboas J. M. Williams J. M.. (2005). Engineering craniofacial scaffolds. Orthod. Craniofac. Res. 8, 162–173. 10.1111/j.1601-6343.2005.00329.x 16022718

[B71] Hossfeld S. Nolte A. Hartmann H. Recke M. Schaller M. Walker T.. (2013). Bioactive coronary stent coating based on layer-by-layer technology for siRNA release. Acta Biomater. 9, 6741–6752. 10.1016/j.actbio.2013.01.013 23333865

[B72] Hou J. Wang J. Cao L. Qian X. Xing W. Lu J.. (2012). Segmental bone regeneration using rhBMP-2-loaded collagen/chitosan microspheres composite scaffold in a rabbit model. Biomed. Mater. 7:035002. 10.1088/1748-6041/7/3/035002 22358865

[B73] Huang X. Brazel C. S. (2001). On the importance and mechanisms of burst release in matrix-controlled drug delivery systems. J. Control. Release 73, 121–136. 10.1016/S0168-3659(01)00248-6 11516493

[B74] Hulbert S. F. Young F. A. Mathews R. S. Klawitter J. J. Talbert C. D. Stelling F. H. (1970). Potential of ceramic materials as permanently implantable skeletal prostheses. J. Biomed. Mater. Res. 4, 433–456. 10.1002/jbm.820040309 5469185

[B75] Hyzy S. L. Olivares-Navarrete R. Schwartz Z. Boyan B. D. (2012). BMP2 induces osteoblast apoptosis in a maturation state and noggin-dependent manner. J. Cell. Biochem. 113, 3236–3245. 10.1002/jcb.24201 22628200 PMC3852427

[B76] Iemura S. Yamamoto T. S. Takagi C. Uchiyama H. Natsume T. Shimasaki S.. (1998). Direct binding of follistatin to a complex of bone-morphogenetic protein and its receptor inhibits ventral and epidermal cell fates in early Xenopus embryo. Proc. Natl. Acad. Sci. U.S.A. 95, 9337–9342. 10.1073/pnas.95.16.9337 9689081 PMC21339

[B77] Ifkovits J. L. Devlin J. J. Eng G. Martens T. P. Vunjak-Novakovic G. Burdick J. A. (2009). Biodegradable fibrous scaffolds with tunable properties formed from photo-cross-linkable poly(glycerol sebacate). ACS Appl. Mater. Interfaces 1, 1878–1886. 10.1021/am900403k 20160937 PMC2765054

[B78] Ishisaki A. Yamato K. Hashimoto S. Nakao A. Tamaki K. Nonaka K.. (1999). Differential inhibition of Smad6 and Smad7 on bone morphogenetic protein- and activin-mediated growth arrest and apoptosis in B cells. J. Biol. Chem. 274, 13637–13642. 10.1074/jbc.274.19.13637 10224135

[B79] Jensen L. N. Sturup J. Kramhoft M. Jensen J. S. (1991). Histological evaluation of cortical bone reaction to PMMA cement. Acta Orthop. Belg. 57, 254–259. 1950508

[B80] Jeon O. Powell C. Solorio L. D. Krebs M. D. Alsberg E. (2011). Affinity-based growth factor delivery using biodegradable, photocrosslinked heparin-alginate hydrogels. J. Control. Release 154, 258–266. 10.1016/j.jconrel.2011.06.027 21745508 PMC3541683

[B81] Jin Y. Kundu B. Cai Y. Kundu S. C. Yao J. (2015). Bio-inspired mineralization of hydroxyapatite in 3D silk fibroin hydrogel for bone tissue engineering. Colloids Surf. B Biointerfaces 134, 339–345. 10.1016/j.colsurfb.2015.07.015 26209967

[B82] Kalfas I. H. (2001). Principles of bone healing. Neurosurg. Focus 10:E1. 10.3171/foc.2001.10.4.2 16732625

[B83] Kanellakopoulou K. Giamarellos-Bourboulis E. J. (2000). Carrier systems for the local delivery of antibiotics in bone infections. Drugs 59, 1223–1232. 10.2165/00003495-200059060-00003 10882159

[B84] Karageorgiou V. Tomkins M. Fajardo R. Meinel L. Snyder B. Wade K. (2006). Porous silk fibroin 3-D scaffolds for delivery of bone morphogenetic protein-2 *in vitro* and *in vivo*. J. Biomed. Mater. Res. A 78, 324–334. 10.1002/jbm.a.30728 16637042

[B85] Karp J. M. Sarraf F. Shoichet M. S. Davies J. E. (2004). Fibrin-filled scaffolds for bone-tissue engineering: an *in vivo* study. J. Biomed. Mater. Res. A 71, 162–171. 10.1002/jbm.a.30147 15368266

[B86] Katagiri T. Yamaguchi A. Komaki M. Abe E. Takahashi N. Ikeda T.. (1994). Bone morphogenetic protein-2 converts the differentiation pathway of C2C12 myoblasts into the osteoblast lineage. J. Cell Biol. 127, 1755–1766. 10.1083/jcb.127.6.1755 7798324 PMC2120318

[B87] Kato S. Sangadala S. Tomita K. Titus L. Boden S. D. (2011). A synthetic compound that potentiates bone morphogenetic protein-2-induced transdifferentiation of myoblasts into the osteoblastic phenotype. Mol. Cell. Biochem. 349, 97–106. 10.1007/s11010-010-0664-6 21110071 PMC3043116

[B88] Katoh K. Tanabe T. Yamauchi K. (2004). Novel approach to fabricate keratin sponge scaffolds with controlled pore size and porosity. Biomaterials 25, 4255–4262. 10.1016/j.biomaterials.2003.11.018 15046915

[B89] Kempen D. H. Lu L. Hefferan T. E. Creemers L. B. Maran A. Classic K. L.. (2008). Retention of *in vitro* and *in vivo* BMP-2 bioactivities in sustained delivery vehicles for bone tissue engineering. Biomaterials 29, 3245–3252. 10.1016/j.biomaterials.2008.04.031 18472153 PMC2577841

[B90] Kirchhof K. Groth T. (2008). Surface modification of biomaterials to control adhesion of cells. Clin. Hemorheol. Microcirc. 39, 247–251. 10.3233/CH-2008-1089 18503133

[B91] Kirker-Head C. Karageorgiou V. Hofmann S. Fajardo R. Betz O. Merkle H. P.. (2007). BMP-silk composite matrices heal critically sized femoral defects. Bone 41, 247–255. 10.1016/j.bone.2007.04.186 17553763 PMC2695963

[B92] Klemm K. W. (1993). Antibiotic bead chains. Clin. Orthop. Relat. Res. 295, 63–76. 10.1097/00003086-199310000-00011 8403672

[B93] Kowalczewski C. (2014). Natural Polymeric Carriers for Local Delivery of Therapeutic Agents to Promote Enhanced Bone Regeneration, Biomedical Engineering. Wake Forest University, 190.

[B94] Kowalczewski C. J. Saul J. M. (2015). Surface-mediated delivery of siRNA from fibrin hydrogels for knockdown of the BMP-2 binding antagonist noggin. Acta Biomater. 25, 109–120. 10.1016/j.actbio.2015.07.045 26234488 PMC4562886

[B95] Kowalczewski C. J. Tombyln S. Wasnick D. C. Hughes M. R. Ellenburg M. D. Callahan M. F.. (2014). Reduction of ectopic bone growth in critically-sized rat mandible defects by delivery of rhBMP-2 from kerateine biomaterials. Biomaterials 35, 3220–3228. 10.1016/j.biomaterials.2013.12.087 24439399 PMC4321825

[B96] Krebs M. D. Jeon O. Alsberg E. (2009). Localized and sustained delivery of silencing RNA from macroscopic biopolymer hydrogels. J. Am. Chem. Soc. 131, 9204–9206. 10.1021/ja9037615 19530653

[B97] Laurencin C. T. Ashe K. M. Henry N. Kan H. M. Lo K. W. (2014). Delivery of small molecules for bone regenerative engineering: preclinical studies and potential clinical applications. Drug Discov. Today 19, 794–800. 10.1016/j.drudis.2014.01.012 24508820 PMC4048776

[B98] Lawson M. A. Barralet J. E. Wang L. Shelton R. M. Triffitt J. T. (2004). Adhesion and growth of bone marrow stromal cells on modified alginate hydrogels. Tissue Eng. 10, 1480–1491. 10.1089/ten.2004.10.1480 15588407

[B99] Le Guehennec L. Layrolle P. Daculsi G. (2004). A review of bioceramics and fibrin sealant. Eur. Cell. Mater. 8, 1–10; discussion: 10-1. 10.22203/eCM.v008a01 15494929

[B100] Li B. Brown K. V. Wenke J. C. Guelcher S. A. (2010). Sustained release of vancomycin from polyurethane scaffolds inhibits infection of bone wounds in a rat femoral segmental defect model. J. Control. Release 145, 221–230. 10.1016/j.jconrel.2010.04.002 20382191

[B101] Li C. Vepari C. Jin H. J. Kim H. J. Kaplan D. L. (2006). Electrospun silk-BMP-2 scaffolds for bone tissue engineering. Biomaterials 27, 3115–3124. 10.1016/j.biomaterials.2006.01.022 16458961

[B102] Li H. Chang J. (2005). Preparation, characterization and *in vitro* release of gentamicin from PHBV/wollastonite composite microspheres. J. Control. Release 107, 463–473. 10.1016/j.jconrel.2005.05.019 16154657

[B103] Li H. Ji Q. Chen X. Sun Y. Xu Q. Deng P.. (2017). Accelerated bony defect healing based on chitosan thermosensitive hydrogel scaffolds embedded with chitosan nanoparticles for the delivery of BMP2 plasmid DNA. J. Biomed. Mater. Res. A 105, 265–273. 10.1002/jbm.a.35900 27636714

[B104] Li J. Hong J. Zheng Q. Guo X. Lan S. Cui F. (2011). Repair of rat cranial bone defects with nHAC/PLLA and BMP-2-related peptide or rhBMP-2. J. Orthop. Res. 29, 1745–1752. 10.1002/jor.21439 21500252

[B105] Lieb E. Tessmar J. Hacker M. Fischbach C. Rose D. Blunk T.. (2003). Poly(D,L-lactic acid)-poly(ethylene glycol)-monomethyl ether diblock copolymers control adhesion and osteoblastic differentiation of marrow stromal cells. Tissue Eng. 9, 71–84. 10.1089/107632703762687555 12625956

[B106] Lim F. Sun A. M. (1980). Microencapsulated islets as bioartificial endocrine pancreas. Science 210, 908–910. 6776628 10.1126/science.6776628

[B107] Lind M. Schumacker B. Soballe K. Keller J. Melsen F. Bunger C. (1993). Transforming growth factor-beta enhances fracture healing in rabbit tibiae. Acta Orthop. Scand. 64, 553–556. 8237323 10.3109/17453679308993691

[B108] Lowman A. M. Peppas N. A. (1999). Hydrogels, in Encyclopedia of Controlled Drug Delivery, ed. Mathiowitz E. (New York, NY: Wiley), 397–418.

[B109] Lu H. Kawazoe N. Kitajima T. Myoken Y. Tomita M. Umezawa A.. (2012). Spatial immobilization of bone morphogenetic protein-4 in a collagen-PLGA hybrid scaffold for enhanced osteoinductivity. Biomaterials 33, 6140–6146. 10.1016/j.biomaterials.2012.05.038 22698726

[B110] Luna S. M. Silva S. S. Gomes M. E. Mano J. F. Reis R. L. (2011). Cell adhesion and proliferation onto chitosan-based membranes treated by plasma surface modification. J. Biomater. Appl. 26, 101–116. 10.1177/0885328210362924 20511386

[B111] Ma D. An G. Liang M. Liu Y. Zhang B. Wang Y. (2016). A composited PEG-silk hydrogel combining with polymeric particles delivering rhBMP-2 for bone regeneration. Mater. Sci. Eng C 65, 221–231. 10.1016/j.msec.2016.04.043 27157747

[B112] Ma Z. Yang C. Song W. Wang Q. Kjems J. Gao S. (2014). Chitosan hydrogel as siRNA vector for prolonged gene silencing. J. Nanobiotechnol. 12:23 10.1186/1477-3155-12-23 PMC4104730 24946934

[B113] Maloney W. J. Jasty M. Rosenberg A. Harris W. H. (1990). Bone lysis in well-fixed cemented femoral components. J. Bone Joint Surg. Br. 72, 966–970. 10.1302/0301-620X.72B6.2246299 2246299

[B114] Mandal B. B. Grinberg A. Gil E. S. Panilaitis B. Kaplan D. L. (2012). High-strength silk protein scaffolds for bone repair. Proc. Natl. Acad. Sci. U.S.A. 109, 7699–7704. 10.1073/pnas.1119474109 22552231 PMC3356671

[B115] Mano J. F. Silva G. A. Azevedo H. S. Malafaya P. B. Sousa R. A. Silva S. S.. (2007). Natural origin biodegradable systems in tissue engineering and regenerative medicine: present status and some moving trends. J. R. Soc. Interface 4, 999–1030. 10.1098/rsif.2007.0220 17412675 PMC2396201

[B116] Marolt D. Augst A. Freed L. E. Vepari C. Fajardo R. Patel N.. (2006). Bone and cartilage tissue constructs grown using human bone marrow stromal cells, silk scaffolds and rotating bioreactors. Biomaterials 27, 6138–6149. 10.1016/j.biomaterials.2006.07.015 16895736

[B117] Martello F. Tocchio A. Tamplenizza M. Gerges I. Pistis V. Recenti R.. (2014). Poly(amido-amine)-based hydrogels with tailored mechanical properties and degradation rates for tissue engineering. Acta Biomater. 10, 1206–1215. 10.1016/j.actbio.2013.12.023 24361426

[B118] Martino M. M. Briquez P. S. Guc E. Tortelli F. Kilarski W. W. Metzger S.. (2014). Growth factors engineered for super-affinity to the extracellular matrix enhance tissue healing. Science 343, 885–888. 10.1126/science.1247663 24558160

[B119] Martino M. M. Hubbell J. A. (2010). The 12th-14th type III repeats of fibronectin function as a highly promiscuous growth factor-binding domain. FASEB J. 24, 4711–4721. 10.1096/fj.09-151282 20671107

[B120] Martino M. M. Mochizuki M. Rothenfluh D. A. Rempel S. A. Hubbell J. A. Barker T. H. (2009). Controlling integrin specificity and stem cell differentiation in 2D and 3D environments through regulation of fibronectin domain stability. Biomaterials 30, 1089–1097. 10.1016/j.biomaterials.2008.10.047 19027948 PMC2718049

[B121] McGill M. Coburn J. M. Partlow B. P. Mu X. Kaplan D. L. (2017). Molecular and macro-scale analysis of enzyme-crosslinked silk hydrogels for rational biomaterial design. Acta Biomater. 63, 76–84. 10.1016/j.actbio.2017.09.020 28919509

[B122] McKay W. F. Peckham S. M. Badura J. M. (2007). A comprehensive clinical review of recombinant human bone morphogenetic protein-2 (INFUSE Bone Graft). Int. Orthop. 31, 729–734. 10.1007/s00264-007-0418-6 17639384 PMC2266665

[B123] Mehta M. Schmidt-Bleek K. Duda G. N. Mooney D. J. (2012). Biomaterial delivery of morphogens to mimic the natural healing cascade in bone. Adv. Drug Deliv. Rev. 64, 1257–1276. 10.1016/j.addr.2012.05.006 22626978 PMC3425736

[B124] Meinel L. Fajardo R. Hofmann S. Langer R. Chen J. Snyder B.. (2005). Silk implants for the healing of critical size bone defects. Bone 37, 688–698. 10.1016/j.bone.2005.06.010 16140599

[B125] Midha S. Murab S. Ghosh S. (2016). Osteogenic signaling on silk-based matrices. Biomaterials 97, 133–153. 10.1016/j.biomaterials.2016.04.020 27163625

[B126] Mittnacht U. Hartmann H. Hein S. Oliveira H. Dong M. Pego A. P.. (2010). Chitosan/siRNA nanoparticles biofunctionalize nerve implants and enable neurite outgrowth. Nano Lett. 10, 3933–3939. 10.1021/nl1016909 20795625

[B127] Mosesson M. W. Sherry S. (1966). The preparation and properties of human fibrinogen of relatively high solubility. Biochemistry 5, 2829–2835. 10.1021/bi00873a008 5961868

[B128] Murakami G. Watabe T. Takaoka K. Miyazono K. Imamura T. (2003). Cooperative inhibition of bone morphogenetic protein signaling by Smurf1 and inhibitory Smads. Mol. Biol. Cell 14, 2809–2817. 10.1091/mbc.e02-07-0441 12857866 PMC165678

[B129] Murphy C. M. Haugh M. G. O'Brien F. J. (2010). The effect of mean pore size on cell attachment, proliferation and migration in collagen-glycosaminoglycan scaffolds for bone tissue engineering. Biomaterials 31, 461–466. 10.1016/j.biomaterials.2009.09.063 19819008

[B130] Nafee N. Zewail M. Boraie N. (2017). Alendronate-loaded, biodegradable smart hydrogel: a promising injectable depot formulation for osteoporosis. J. Drug Target. 10.1080/1061186X.2017.1390670 [Epub ahead of print]. 29073792

[B131] Nakamura T. Hara Y. Tagawa M. Tamura M. Yuge T. Fukuda H.. (1998). Recombinant human basic fibroblast growth factor accelerates fracture healing by enhancing callus remodeling in experimental dog tibial fracture. J. Bone Miner. Res. 13, 942–949. 10.1359/jbmr.1998.13.6.942 9626625

[B132] Naskar D. Nayak S. Dey T. Kundu S. C. (2014). Non-mulberry silk fibroin influence osteogenesis and osteoblast-macrophage cross talk on titanium based surface. Sci. Rep. 4:4745. 10.1038/srep04745 24752225 PMC3994497

[B133] Nerem R. M. (2006). Tissue engineering: the hope, the hype, and the future. Tissue Eng. 12, 1143–1150. 10.1089/ten.2006.12.1143 16771630

[B134] Nevins M. Kirker-Head C. Wozney J. A. Palmer R. Graham D. (1996). Bone formation in the goat maxillary sinus induced by absorbable collagen sponge implants impregnated with recombinant human bone morphogenetic protein-2. Int. J. Periodontics Restor. Dent. 16, 8–19. 8631615

[B135] Nguyen H. Morgan D. A. Forwood M. R. (2007). Sterilization of allograft bone: is 25 kGy the gold standard for gamma irradiation? Cell Tissue Bank 8, 81–91. 10.1007/s10561-006-9019-7 16821106

[B136] Nguyen M. K. Jeon O. Krebs M. D. Schapira D. Alsberg E. (2014). Sustained localized presentation of RNA interfering molecules from in situ forming hydrogels to guide stem cell osteogenic differentiation. Biomaterials 35, 6278–6286. 10.1016/j.biomaterials.2014.04.048 24831973 PMC4157362

[B137] Niederwanger M. Urist M. R. (1996). Demineralized bone matrix supplied by bone banks for a carrier of recombinant human bone morphogenetic protein (rhBMP-2): a substitute for autogeneic bone grafts. J. Oral Implantol. 22, 210–215. 9524496

[B138] Niu X. Fan Y. Liu X. Li X. Li P. Wang J.. (2011). Repair of bone defect in femoral condyle using microencapsulated chitosan, nanohydroxyapatite/collagen and poly(L-lactide)-based microsphere-scaffold delivery system. Artif. Organs 35, E119–E128. 10.1111/j.1525-1594.2011.01274.x 21658081

[B139] Niu X. Feng Q. Wang M. Guo X. Zheng Q. (2009). Porous nano-HA/collagen/PLLA scaffold containing chitosan microspheres for controlled delivery of synthetic peptide derived from BMP-2. J. Control. Release 134, 111–117. 10.1016/j.jconrel.2008.11.020 19100794

[B140] Normile D. (2009). Insect genetics. Sequencing 40 silkworm genomes unravels history of cultivation. Science 325, 1058–1059. 10.1126/science.325_1058a 19713499

[B141] Ochi K. Chen G. Ushida T. Gojo S. Segawa K. Tai H.. (2003). Use of isolated mature osteoblasts in abundance acts as desired-shaped bone regeneration in combination with a modified poly-DL-lactic-co-glycolic acid (PLGA)-collagen sponge. J. Cell. Physiol. 194, 45–53. 10.1002/jcp.10185 12447988

[B142] Oka S. (1981). Cardiovascular Hemorheology. Cambridge, UK: Press Syndicate of the University of Cambridge.

[B143] Okamoto M. Murai J. Yoshikawa H. Tsumaki N. (2006). Bone morphogenetic proteins in bone stimulate osteoclasts and osteoblasts during bone development. J. Bone Miner. Res. 21, 1022–1033. 10.1359/jbmr.060411 16813523

[B144] Panda N. Bissoyi A. Pramanik K. Biswas A. (2015). Development of novel electrospun nanofibrous scaffold from P. Ricini And A. Mylitta silk fibroin blend with improved surface and biological properties. Mater. Sci. Eng. C 48, 521–532. 10.1016/j.msec.2014.12.010 25579953

[B145] Papadimitriou K. Karkavelas G. Vouros I. Kessopoulou E. Konstantinidis A. (2015). Effects of local application of simvastatin on bone regeneration in femoral bone defects in rabbit. J. Craniomaxillofac. Surg. 43, 232–237. 10.1016/j.jcms.2014.11.011 25534041

[B146] Park K. H. Kim H. Moon S. Na K. (2009). Bone morphogenic protein-2 (BMP-2) loaded nanoparticles mixed with human mesenchymal stem cell in fibrin hydrogel for bone tissue engineering. J. Biosci. Bioeng. 108, 530–537. 10.1016/j.jbiosc.2009.05.021 19914589

[B147] Partlow B. P. Hanna C. W. Rnjak-Kovacina J. Moreau J. E. Applegate M. B. Burke K. A.. (2014). Highly tunable elastomeric silk biomaterials. Adv. Funct. Mater. 24, 4615–4624. 10.1002/adfm.201400526 25395921 PMC4225629

[B148] Patel R. A. Wilson R. F. Patel P. A. Palmer R. M. (2013). The effect of smoking on bone healing: a systematic review. Bone Joint Res. 2, 102–111. 10.1302/2046-3758.26.2000142 23836474 PMC3686151

[B149] Patel Z. S. Young S. Tabata Y. Jansen J. A. Wong M. E. Mikos A. G. (2008). Dual delivery of an angiogenic and an osteogenic growth factor for bone regeneration in a critical size defect model. Bone 43, 931–940. 10.1016/j.bone.2008.06.019 18675385 PMC3014108

[B150] Pathi S. P. Kowalczewski C. Tadipatri R. Fischbach C. (2010). A novel 3-D mineralized tumor model to study breast cancer bone metastasis. PLoS ONE 5:e8849. 10.1371/journal.pone.0008849 20107512 PMC2809751

[B151] Pera M. F. Andrade J. Houssami S. Reubinoff B. Trounson A. Stanley E. G.. (2004). Regulation of human embryonic stem cell differentiation by BMP-2 and its antagonist noggin. J. Cell Sci. 117, 1269–1280. 10.1242/jcs.00970 14996946

[B152] Percival E. (1979). Polysaccharides of green, red and brown seaweeds - their basic structure, biosynthesis and function. Br. Phycol. J. 14, 103–117. 10.1080/00071617900650121

[B153] Pierschbacher M. D. Ruoslahti E. (1984). Cell attachment activity of fibronectin can be duplicated by small synthetic fragments of the molecule. Nature 309, 30–33. 10.1038/309030a0 6325925

[B154] Pillay V. Danckwerts M. P. Muhidinov Z. Fassihi R. (2005). Novel modulation of drug delivery using binary zinc-alginate-pectinate polyspheres for zero-order kinetics over several days: Experimental design strategy to elucidate the crosslinking mechanism. Drug Dev. Ind. Pharm. 31, 191–207. 10.1081/DDC-200047806 15773286

[B155] Plowman J. E. Deb-Choudhury S. Thomas A. Clerens S. Cornellison C. D. Grosvenor A. J.. (2010). Characterisation of low abundance wool proteins through novel differential extraction techniques. Electrophoresis 31, 1937–1946. 10.1002/elps.201000053 20564690

[B156] Polo-Corrales L. Latorre-Esteves M. Ramirez-Vick J. E. (2014). Scaffold design for bone regeneration. J. Nanosci. Nanotechnol. 14, 15–56. 10.1166/jnn.2014.9127 24730250 PMC3997175

[B157] Profumo A. Turci M. Damonte G. Ferri F. Magatti D. Cardinali B.. (2003). Kinetics of fibrinopeptide release by thrombin as a function of CaCl2 concentration: different susceptibility of FPA and FPB and evidence for a fibrinogen isoform-specific effect at physiological Ca2+ concentration. Biochemistry 42, 12335–12348. 10.1021/bi034411e 14567695

[B158] Puska M. Aho A. J. Vallittu P. (2011). Polymer composites for bone reconstruction, in Advances in Composite Materials - Analysis of Natural and Man-Made Materials, ed. Tesinova P. (Rijeka: InTech), 55–72.

[B159] Qi Y. Zhao T. Yan W. Xu K. Shi Z. Wang J. (2013). Mesenchymal stem cell sheet transplantation combined with locally released simvastatin enhances bone formation in a rat tibia osteotomy model. Cytotherapy 15, 44–56. 10.1016/j.jcyt.2012.10.006 23260085

[B160] Qiu Y. Lim J. J. Scott L. Jr. Adams R. C. Bui H. T. Temenoff J. S. (2011). PEG-based hydrogels with tunable degradation characteristics to control delivery of marrow stromal cells for tendon overuse injuries. Acta Biomater. 7, 959–966. 10.1016/j.actbio.2010.11.002 21056127

[B161] Qu H. Guo W. Yang R. Li D. Tang S. Yang Y. (2015). Reconstruction of segmental bone defect of long bones after tumor resection by devitalized tumor-bearing bone. World J. Surg. Oncol. 13:282 10.1186/s12957-015-0694-3 26399398 PMC4581416

[B162] Rahman M. S. Akhtar N. Jamil H. M. Banik R. S. Asaduzzaman S. M. (2015). TGF-beta/BMP signaling and other molecular events: regulation of osteoblastogenesis and bone formation. Bone Res. 3:15005. 10.1038/boneres.2015.5 26273537 PMC4472151

[B163] Rajangam T. An S. S. (2013). Fibrinogen and fibrin based micro and nano scaffolds incorporated with drugs, proteins, cells and genes for therapeutic biomedical applications. Int. J. Nanomedicine 8, 3641–3662. 10.2147/IJN.S43945 24106425 PMC3792008

[B164] Razak S. I. A. Sharif N. F. A. Rahman W. A. (2012). Biodegradable polymers and their bone applications: a review. IJBAS-IJENS 12, 31–49.

[B165] Reichl S. (2009). Films based on human hair keratin as substrates for cell culture and tissue engineering. Biomaterials 30, 6854–6866. 10.1016/j.biomaterials.2009.08.051 19783297

[B166] Rodriguez-Evora M. Garcia-Pizarro E. del Rosario C. Perez-Lopez J. Reyes R. Delgado A.. (2014). Smurf1 knocked-down, mesenchymal stem cells and BMP-2 in an electrospun system for bone regeneration. Biomacromolecules 15, 1311–1322. 10.1021/bm401854d 24559435

[B167] Rowe N. M. Mehrara B. J. Luchs J. S. Dudziak M. E. Steinbrech D. S. Illei P. B.. (1999). Angiogenesis during mandibular distraction osteogenesis. Ann. Plast. Surg. 42, 470–475. 10.1097/00000637-199905000-00002 10340853

[B168] Rowe S. L. Lee S. Stegemann J. P. (2007). Influence of thrombin concentration on the mechanical and morphological properties of cell-seeded fibrin hydrogels. Acta Biomater. 3, 59–67. 10.1016/j.actbio.2006.08.006 17085089 PMC1852453

[B169] Saha S. Pal S. (1984). Mechanical properties of bone cement: a review. J. Biomed. Mater. Res. 18, 435–462. 10.1002/jbm.820180411 6376513

[B170] Sahu N. Baligar P. Midha S. Kundu B. Bhattacharjee M. Mukherjee S.. (2015). Nonmulberry silk fibroin scaffold shows superior osteoconductivity than mulberry silk fibroin in calvarial bone regeneration. Adv. Healthc. Mater. 4, 1709–1721. 10.1002/adhm.201500283 26084249

[B171] Saito A. Suzuki Y. Ogata S. Ohtsuki C. Tanihara M. (2004). Prolonged ectopic calcification induced by BMP-2-derived synthetic peptide. J. Biomed. Mater. Res. A 70, 115–121. 10.1002/jbm.a.30071 15174115

[B172] Saltzman W. M. (2001). Drug Delivery : Engineering Principles for Drug Therapy. Oxford; New York, NY: Oxford University Press.

[B173] Sanzana E. S. Navarro M. Ginebra M. P. Planell J. A. Ojeda A. C. Montecinos H. A. (2014). Role of porosity and pore architecture in the *in vivo* bone regeneration capacity of biodegradable glass scaffolds. J. Biomed. Mater. Res. A 102, 1767–1773. 10.1002/jbm.a.34845 23813739

[B174] Saul J. M. Ellenburg M. D. de Guzman R. C. Van Dyke M. (2011). Keratin hydrogels support the sustained release of bioactive ciprofloxacin. J. Biomed. Mater. Res. Part A 98, 544–553. 10.1002/jbm.a.33147 21681948

[B175] Saul J. M. Linnes M. P. Ratner B. D. Giachelli C. M. Pun S. H. (2007). Delivery of non-viral gene carriers from sphere-templated fibrin scaffolds for sustained transgene expression. Biomaterials 28, 4705–4716. 10.1016/j.biomaterials.2007.07.026 17675152

[B176] Schimandle J. H. Boden S. D. Hutton W. C. (1995). Experimental spinal fusion with recombinant human bone morphogenetic protein-2. Spine 20, 1326–1337. 10.1097/00007632-199520120-00002 7676329

[B177] Schmidmaier G. Schwabe P. Wildemann B. Haas N. P. (2007). Use of bone morphogenetic proteins for treatment of non-unions and future perspectives. Injury 38(Suppl. 4), S35–S41. 10.1016/S0020-1383(08)70007-X 18224735

[B178] Schmidmaier G. Wildemann B. Gabelein T. Heeger J. Kandziora F. Haas N. P.. (2003). Synergistic effect of IGF-I and TGF-beta1 on fracture healing in rats: single versus combined application of IGF-I and TGF-beta1. Acta Orthop. Scand. 74, 604–610. 10.1080/00016470310018036 14620984

[B179] Schmitz J. P. Hollinger J. O. (1986). The critical size defect as an experimental model for craniomandibulofacial nonunions. Clin. Orthop. Relat. Res. 205, 299–308. 10.1097/00003086-198604000-00036 3084153

[B180] Schoelles K. Snyder D. Kaczmarek J. Kuserk E. Erinoff E. Turkelson C. (2005). The Role of Bone Growth Stimulating Devices and Orthobiologics in Healing Nonunion Fractures. Rockville, MD: Agency for Healthcare Research and Quality (US). 25879121

[B181] Seekamp A. Köntopp H. Schandelmaier P. Krettek C. Tscherne H. (2000). Bacterial cultures and bacterial infection in open fractures. Eur. J. Trauma 26, 131–138. 10.1007/s000680050011

[B182] Senior R. M. Skogen W. F. Griffin G. L. Wilner G. D. (1986). Effects of fibrinogen derivatives upon the inflammatory response. Studies with human fibrinopeptide B. J. Clin. Invest. 77, 1014–1019. 10.1172/JCI112353 3005361 PMC423507

[B183] Shah H. Rousset M. Canavese F. (2012). Congenital pseudarthrosis of the tibia: management and complications. Indian J. Orthop. 46, 616–626. 10.4103/0019-5413.104184 23325962 PMC3543877

[B184] Shats E. A. Nair C. H. Dhall D. P. (1997). Interaction of endothelial cells and fibroblasts with modified fibrin networks: role in atherosclerosis. Atherosclerosis 129, 9–15. 10.1016/S0021-9150(96)06003-0 9069511

[B185] Shen H. Hu X. Yang F. Bei J. Wang S. (2009). The bioactivity of rhBMP-2 immobilized poly(lactide-co-glycolide) scaffolds. Biomaterials 30, 3150–3157. 10.1016/j.biomaterials.2009.02.004 19232709

[B186] Shi M. Kretlow J. D. Nguyen A. Young S. Scott Baggett L. Wong M. E.. (2010). Antibiotic-releasing porous polymethylmethacrylate constructs for osseous space maintenance and infection control. Biomaterials 31, 4146–4156. 10.1016/j.biomaterials.2010.01.112 20153893 PMC2839066

[B187] Shi Z. Neoh K. G. Kang E. T. Wang W. (2006). Antibacterial and mechanical properties of bone cement impregnated with chitosan nanoparticles. Biomaterials 27, 2440–2449. 10.1016/j.biomaterials.2005.11.036 16338001

[B188] Sokolsky-Papkov M. Agashi K. Olaye A. Shakesheff K. Domb A. J. (2007). Polymer carriers for drug delivery in tissue engineering. Adv. Drug Deliv. Rev. 59, 187–206. 10.1016/j.addr.2007.04.001 17540473

[B189] Spector J. A. Luchs J. S. Mehrara B. J. Greenwald J. A. Smith L. P. Longaker M. T. (2001). Expression of bone morphogenetic proteins during membranous bone healing. Plast. Reconstr. Surg. 107, 124–134. 10.1097/00006534-200101000-00018 11176610

[B190] Spicer P. P. Kretlow J. D. Young S. Jansen J. A. Kasper F. K. Mikos A. G. (2012). Evaluation of bone regeneration using the rat critical size calvarial defect. Nat. Protoc. 7, 1918–1929. 10.1038/nprot.2012.113 23018195 PMC3513397

[B191] Sporn L. A. Bunce L. A. Francis C. W. (1995). Cell proliferation on fibrin: modulation by fibrinopeptide cleavage. Blood 86, 1802–1810. 7655010

[B192] Srinivasan B. Kumar R. Shanmugam K. Sivagnam U. T. Reddy N. P. Sehgal P. K. (2010). Porous keratin scaffold-promising biomaterial for tissue engineering and drug delivery. J. Biomed. Mater. Res. Part B Appl. Biomater. 92, 5–12. 10.1002/jbm.b.31483 19637379

[B193] Stadelmann V. A. Gauthier O. Terrier A. Bouler J. M. Pioletti D. P. (2008). Implants delivering bisphosphonate locally increase periprosthetic bone density in an osteoporotic sheep model. A pilot study. Eur. Cell. Mater. 16, 10–16. 18671203 10.22203/ecm.v016a02

[B194] Stafford D. A. Brunet L. J. Khokha M. K. Economides A. N. Harland R. M. (2011). Cooperative activity of noggin and gremlin 1 in axial skeleton development. Development 138, 1005–1014. 10.1242/dev.051938 21303853 PMC3035099

[B195] Suganuma J. Alexander H. (1993). Biological response of intramedullary bone to poly-L-lactic acid. J. Appl. Biomater. 4, 13–27. 10.1002/jab.770040103

[B196] Suliman S. Xing Z. Wu X. Xue Y. Pedersen T. O. Sun Y.. (2015). Release and bioactivity of bone morphogenetic protein-2 are affected by scaffold binding techniques *in vitro* and *in vivo*. J. Control. Release 197, 148–157. 10.1016/j.jconrel.2014.11.003 25445698

[B197] Taboas J. M. Maddox R. D. Krebsbach P. H. Hollister S. J. (2003). Indirect solid free form fabrication of local and global porous, biomimetic and composite 3D polymer-ceramic scaffolds. Biomaterials 24, 181–194. 10.1016/S0142-9612(02)00276-4 12417192

[B198] Tachibana A. Furuta Y. Takeshima H. Tanabe T. Yamauchi K. (2002). Fabrication of wool keratin sponge scaffolds for long-term cell cultivation. J. Biotechnol. 93, 165–170. 10.1016/S0168-1656(01)00395-9 11738723

[B199] Tachibana A. Kaneko S. Tanabe T. Yamauchi K. (2005). Rapid fabrication of keratin-hydroxyapatite hybrid sponges toward osteoblast cultivation and differentiation. Biomaterials 26, 297–302. 10.1016/j.biomaterials.2004.02.032 15262471

[B200] Tachibana A. Nishikawa Y. Nishino M. Kaneko S. Tanabe T. Yamauchi K. (2006). Modified keratin sponge: binding of bone morphogenetic protein-2 and osteoblast differentiation. J. Biosci. Bioeng. 102, 425–429. 10.1263/jbb.102.425 17189169

[B201] Taormina D. P. Shulman B. S. Karia R. Spitzer A. B. Konda S. R. Egol K. A. (2014). Older age does not affect healing time and functional outcomes after fracture nonunion surgery. Geriatr. Orthop. Surg. Rehabil. 5, 116–121. 10.1177/2151458514532811 25360341 PMC4212425

[B202] Termaat M. F. Den Boer F. C. Bakker F. C. Patka P. Haarman H. J. (2005). Bone morphogenetic proteins. Development and clinical efficacy in the treatment of fractures and bone defects. J. Bone Joint Surg. Am. 87, 1367–1378. 10.2106/JBJS.D.02585 15930551

[B203] Tokatlian T. Shrum C. T. Kadoya W. M. Segura T. (2010). Protease degradable tethers for controlled and cell-mediated release of nanoparticles in 2- and 3-dimensions. Biomaterials 31, 8072–8080. 10.1016/j.biomaterials.2010.07.030 20688389 PMC2945696

[B204] Trice M. E. (2009). Xenograft Risks: What You and Your Patients Need to Know, AAOS Now. Rosemont, IL: American Academy of Orthopaedic Surgeons.

[B205] Tseng S. S. Lee M. A. Reddi A. H. (2008). Nonunions and the potential of stem cells in fracture-healing. J. Bone Joint Surg. Am. 90(Suppl. 1), 92–98. 10.2106/JBJS.G.01192 18292363

[B206] Tsiridis E. Upadhyay N. Giannoudis P. (2007). Molecular aspects of fracture healing: which are the important molecules? Injury 38(Suppl. 1), S11–S25. 10.1016/j.injury.2007.02.006 17383481

[B207] Tsujigiwa H. Nagatsuka H. Lee Y. J. Han P. P. Gunduz M. Legeros R. Z.. (2006). Immobilized rhBMP-2/succinylated type I atelocollagen gene expression of intracellular signaling molecules on ST2 cells. J. Biomed. Mater. Res. A 77, 507–511. 10.1002/jbm.a.30661 16482552

[B208] Urist M. R. (1980). Fundamental and Clinical Bone Physiology. Philadelphia, PA: Lippincott.

[B209] Urist M. R. Strates B. S. (1971). Bone morphogenetic protein. J. Dent. Res. 50, 1392–1406. 10.1177/00220345710500060601 4943222

[B210] Vaibhav B. Nilesh P. Vikram S. Anshul C. (2007). Bone morphogenic protein and its application in trauma cases: a current concept update. Injury 38, 1227–1235. 10.1016/j.injury.2006.12.012 17307180

[B211] Wan D. C. Pomerantz J. H. Brunet L. J. Kim J. B. Chou Y. F. Wu B. M.. (2007). Noggin suppression enhances *in vitro* osteogenesis and accelerates *in vivo* bone formation. J. Biol. Chem. 282, 26450–26459. 10.1074/jbc.M703282200 17609215

[B212] Wang J. C. Alanay A. Mark D. Kanim L. E. Campbell P. A. Dawson E. G.. (2007). A comparison of commercially available demineralized bone matrix for spinal fusion. Eur. Spine J. 16, 1233–1240. 10.1007/s00586-006-0282-x 17205237 PMC2200779

[B213] Wang J. Lu Z. Wientjes M. G. Au J. L. (2010). Delivery of siRNA therapeutics: barriers and carriers. AAPS J. 12, 492–503. 10.1208/s12248-010-9210-4 20544328 PMC2977003

[B214] Wang M. Abbah S. A. Hu T. Toh S. Y. Lam R. W. Goh J. C.. (2013). Minimizing the severity of rhBMP-2-induced inflammation and heterotopic ossification with a polyelectrolyte carrier incorporating heparin on microbead templates. Spine 38, 1452–1458. 10.1097/BRS.0b013e31828a3504 23380826

[B215] Wang R. N. Green J. Wang Z. Deng Y. Qiao M. Peabody M.. (2014). Bone Morphogenetic Protein (BMP) signaling in development and human diseases. Genes Dis. 1, 87–105. 10.1016/j.gendis.2014.07.005 25401122 PMC4232216

[B216] Wang Y. Zhang L. Hu M. Wen W. Xiao H. Niu Y. (2010). Effect of chondroitin sulfate modification on rhBMP-2 release kinetics from collagen delivery system. J. Biomed. Mater. Res. A 92, 693–701. 10.1016/j.biomaterials.2011.03.063 19263491

[B217] Weisel J. W. (2007). Structure of fibrin: impact on clot stability. J. Thrombos. Haemost. 5, 116–124. 10.1111/j.1538-7836.2007.02504.x 17635717

[B218] White L. J. Kirby G. T. Cox H. C. Qodratnama R. Qutachi O. Rose F. R.. (2013). Accelerating protein release from microparticles for regenerative medicine applications. Mater. Sci. Eng. C 33, 2578–2583. 10.1016/j.msec.2013.02.020 23623071 PMC3654200

[B219] Williams C. G. Malik A. N. Kim T. K. Manson P. N. Elisseeff J. H. (2005). Variable cytocompatibility of six cell lines with photoinitiators used for polymerizing hydrogels and cell encapsulation. Biomaterials 26, 1211–1218. 10.1016/j.biomaterials.2004.04.024 15475050

[B220] Woodard J. R. Hilldore A. J. Lan S. K. Park C. J. Morgan A. W. Eurell J. A.. (2007). The mechanical properties and osteoconductivity of hydroxyapatite bone scaffolds with multi-scale porosity. Biomaterials 28, 45–54. 10.1016/j.biomaterials.2006.08.021 16963118

[B221] Wozney J. M. Rosen V. Celeste A. J. Mitsock L. M. Whitters M. J. Kriz R. W.. (1988). Novel regulators of bone formation: molecular clones and activities. Science 242, 1528–1534. 10.1126/science.3201241 3201241

[B222] Wu B. Zheng Q. Guo X. Wu Y. Wang Y. Cui F. (2008). Preparation and ectopic osteogenesis *in vivo* of scaffold based on mineralized recombinant human-like collagen loaded with synthetic BMP-2-derived peptide. Biomed. Mater. 3:044111. 10.1088/1748-6041/3/4/044111 19029602

[B223] Wu M. Chen G. Li Y. P. (2016). TGF-beta and BMP signaling in osteoblast, skeletal development, and bone formation, homeostasis and disease. Bone Res. 4:16009. 10.1038/boneres.2016.9 27563484 PMC4985055

[B224] Xie H. Cui Z. Wang L. Xia Z. Hu Y. Xian L.. (2014). PDGF-BB secreted by preosteoclasts induces angiogenesis during coupling with osteogenesis. Nat. Med. 20, 1270–1278. 10.1038/nm.3668 25282358 PMC4224644

[B225] Xing Z.-C. Yuan J. Chae W.-P. Kang I.-K. KIm S.-Y. (2010). Keratin nanofibers as a bioamterial, in 2010 International Conference on Nanotechnology and Biosensors (Singapore; Hong Kong: IACSIT Press), 120–124.

[B226] Yamamoto M. Takahashi Y. Tabata Y. (2003). Controlled release by biodegradable hydrogels enhances the ectopic bone formation of bone morphogenetic protein. Biomaterials 24, 4375–4383. 10.1016/S0142-9612(03)00337-5 12922150

[B227] Yamauchi K. Hojo H. Yamamoto Y. Tanabe T. (2003). Enhanced cell adhesion on RGDS-carrying keratin film. Mat. Sci. Eng. C-Bio S 23, 467–472. 10.1016/S0928-4931(02)00280-1

[B228] Yang H. S. La W. G. Bhang S. H. Jeon J. Y. Lee J. H. Kim B. S. (2010). Heparin-conjugated fibrin as an injectable system for sustained delivery of bone morphogenetic protein-2. Tissue Eng. Part A 16, 1225–1233. 10.1089/ten.tea.2009.0390 19886733

[B229] Yang H. S. La W. G. Cho Y. M. Shin W. Yeo G. D. Kim B. S. (2012). Comparison between heparin-conjugated fibrin and collagen sponge as bone morphogenetic protein-2 carriers for bone regeneration. Exp. Mol. Med. 44, 350–355. 10.3858/emm.2012.44.5.039 22322342 PMC3366328

[B230] Yoshii T. Hafeman A. E. Esparza J. M. Okawa A. Gutierrez G. Guelcher S. A. (2014). Local injection of lovastatin in biodegradable polyurethane scaffolds enhances bone regeneration in a critical-sized segmental defect in rat femora. J. Tissue Eng. Regen. Med. 8, 589–595. 10.1002/term.1547 22718577

[B231] Young S. Patel Z. S. Kretlow J. D. Murphy M. B. Mountziaris P. M. Baggett L. S.. (2009). Dose effect of dual delivery of vascular endothelial growth factor and bone morphogenetic protein-2 on bone regeneration in a rat critical-size defect model. Tissue Eng. Part A 15, 2347–2362. 10.1089/ten.tea.2008.0510 19249918 PMC2792218

[B232] Zelken J. Wanich T. Gardner M. Griffith M. Bostrom M. (2007). PMMA is superior to hydroxyapatite for colony reduction in induced osteomyelitis. Clin. Orthop. Relat. Res. 462, 190–194. 10.1097/BLO.0b013e3180ca9521 17514008

[B233] Zerhouni E. A. (2005). Translational and clinical science–time for a new vision. N. Engl. J. Med. 353, 1621–1623. 10.1056/NEJMsb053723 16221788

[B234] Zhang H. Migneco F. Lin C. Y. Hollister S. J. (2010). Chemically-conjugated bone morphogenetic protein-2 on three-dimensional polycaprolactone scaffolds stimulates osteogenic activity in bone marrow stromal cells. Tissue Eng. Part A 16, 3441–3448. 10.1089/ten.tea.2010.0132 20560772 PMC2965194

[B235] Zhang Z. K. Li G. Y. Shi B. (2006). Physicochemical properties of collagen, gelatin and collagen hydrolysate derived from bovine limed split wastes. J. Soc. Leath. Tech. Chem. 90, 23–28.

[B236] Zhu M. Wang K. Mei J. Li C. Zhang J. Zheng W.. (2014). Fabrication of highly interconnected porous silk fibroin scaffolds for potential use as vascular grafts. Acta Biomater. 10, 2014–2023. 10.1016/j.actbio.2014.01.022 24486642

